# Health system learning enables generalist neuroimaging models

**DOI:** 10.1038/s41591-026-04497-1

**Published:** 2026-07-10

**Authors:** Akhil Kondepudi, Akshay Rao, Chenhui Zhao, Yiwei Lyu, Samir Harake, Soumyanil Banerjee, Jacob Ogle, Rushikesh Joshi, Anna-Katharina Meissner, Xinhai Hou, Cheng Jiang, Asadur Chowdury, Ashok Srinivasan, Brian Athey, Vikas Gulani, Aditya Pandey, Honglak Lee, Todd Hollon

**Affiliations:** 1 Machine Learning in Neurosurgery Lab, University of Michigan, Ann Arbor, MI USA; 2 University of Michigan Computational Medicine and Bioinformatics, Ann Arbor, MI USA; 3 University of Michigan Computer Science and Engineering, Ann Arbor, MI USA; 4 University of Michigan Neurosurgery, Ann Arbor, MI USA; 5 University of Cologne Neurosurgery, Cologne, Germany; 6 University of Michigan Radiology, Ann Arbor, MI USA

**Keywords:** Machine learning, Translational research, Image processing, Biomedical engineering

## Abstract

Frontier artificial intelligence (AI) models have advanced rapidly through training on internet-scale public data, yet such systems lack access to private clinical data. Neuroimaging is underrepresented in the public domain due to identifiable facial features within magnetic resonance imaging (MRI) and computed tomography (CT) scans, restricting model performance in clinical medicine. Here we show that frontier models underperform on neuroimaging tasks and that learning directly from uncurated data generated during routine clinical care at health systems, a paradigm we call ‘health system learning’, yields high-performance, generalist neuroimaging models. We introduce NeuroVFM, a visual foundation model trained on 5.24 million clinical MRI and CT volumes using a scalable volumetric predictive architecture. NeuroVFM learns comprehensive representations of brain anatomy and pathology, achieving state-of-the-art performance across multiple clinical tasks, including radiologic diagnosis and report generation. The model embeds MRI and CT scans into a shared neuroanatomic latent space and grounds diagnostic findings. When paired with open-source language models, NeuroVFM generates radiology reports that surpass frontier models in accuracy, clinical triage and expert preference. NeuroVFM reduces hallucinated findings and critical errors, offering safer clinical decision support. These results establish health system learning as a paradigm for building generalist medical AI and provide a scalable framework for clinical foundation models.

## Abstract (web summary)

NeuroVFM, trained on routine health system MRI and CT scans, learns general neuroimaging representations that improve diagnosis, report generation and triage, showing how private clinical data will power safer, more accurate medical AI.

## Main

Multimodal large language models (MLLMs) derive much of their capability from learning on internet-scale data, enabling these models to approximate the breadth of human experience across language, images and video. Clinical medicine, however, is underrepresented on the public internet. MLLMs trained exclusively on public data lack access to the rich private information embedded in real-world patient care, which fundamentally limits their performance on clinical tasks. We propose ‘health system learning’ as a new paradigm in which medical foundation models learn directly from uncurated data generated during clinical operations at health systems. By learning in the same complex, nuanced environment in which expert clinicians themselves train, rather than second-hand internet descriptions used by MLLMs, AI models can acquire rich, medical representations grounded in anatomy, pathology and clinical workflows. MLLMs know the map; health system learners know the territory.

To demonstrate the strength of health system learning, we introduce NeuroVFM, a generalist neuroimaging visual foundation model trained on all clinical MRI and CT studies from a large academic health system. Unlike previous models that rely on data curation, human annotations or radiology report supervision^1,2^, NeuroVFM is optimized for general neuroimaging through a self-supervised vision-only algorithm called Volumetric Joint-Embedding Predictive Architectures (Vol-JEPA)^3^. Our method enforces representation learning across imaging modalities and disease spectra, capturing both global and fine-grained neuroanatomic and pathologic features. Health system learning with Vol-JEPA enables NeuroVFM to achieve state-of-the-art performance, surpassing leading proprietary and open-source frontier models across multiple clinical tasks, including radiologic diagnosis and report generation. NeuroVFM predictions are diagnostically grounded, with pathologic image regions mapped to neurologic diagnoses. When integrated with open-source language models, NeuroVFM acts as a visual perception module that outperforms GPT-5 and Claude Sonnet 4.5 on neuroimaging interpretation and triage.

## Learning with Vol-JEPA

**Fig. 1:**
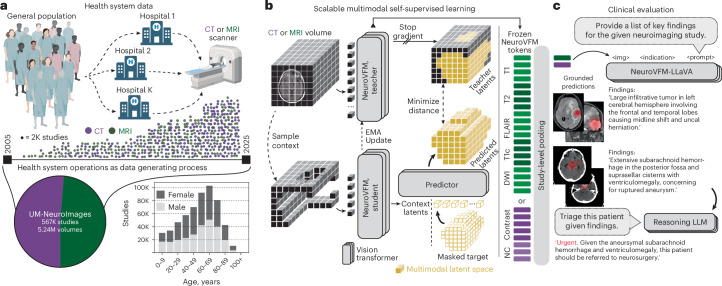
Overview of ‘health system learning’ with NeuroVFM. **a**, Health system learning directly models the data-generating process of clinical operations at large health systems. The UM-NeuroImages dataset comprises 5.24 million volumes from 566,915 studies, acquired over 20 years at Michigan Medicine. Age and sex distributions are shown below. **b**, NeuroVFM was trained using Vol-JEPA, a scalable volumetric self-supervised method that learns a unified latent space for CT and MRI. A 3D volume is partitioned into a small context and larger masked target with the background removed. The context is encoded by an online (‘student’) 3D vision transformer. A predictor combines context latents with position encodings of the masked target region to predict the masked region latents. Ground truth latents for the masked region are generated by an offline (‘teacher’) encoder updated by an exponential moving average, with gradients stopped through the teacher. Training minimizes the distance between the predicted and teacher latents using a smooth L1 loss. **c**, At inference, NeuroVFM encodes all volumes in a neuroimaging study into latent visual tokens for downstream tasks. The same visual tokens can be used to fine-tune an open-source multimodal language model (that is, Qwen3-14B, LLaVA-1.5-style) to generate radiology reports. Illustrative findings and corresponding grounded attention maps are shown. The findings can then be passed to a frontier reasoning LLM (that is, GPT-5-thinking) for interpretation and triage. NC, non-contrast.

**Extended Data Fig. 1:**
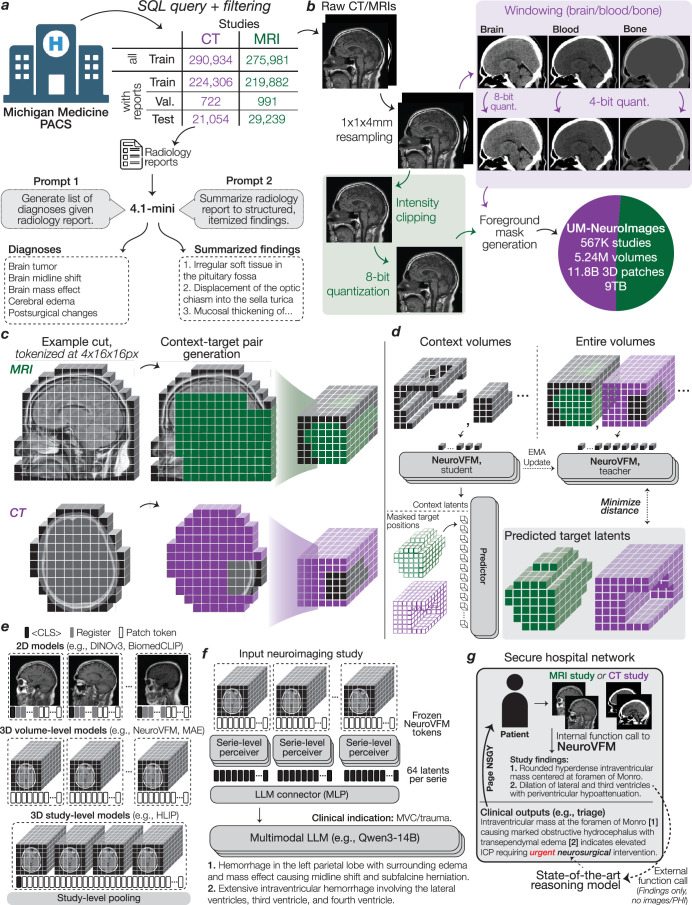
Extended NeuroVFM workflow. **a**, We queried the Michigan Medicine PACS for neuroimaging studies of the head, brain, face, orbits, and neck. After removing non-image and corrupted entries, the UM-NeuroImages cohort comprises 566,915 CT and MRI studies, of which 444,188 have paired radiology reports. Per-modality counts of studies and paired reports for training, validation, and test sets are shown. To obtain training and evaluation labels at scale, we used an LLM report-parsing pipeline ^4^ [ref. ^4^] that (1) extracts a list of expert-defined diagnoses and (2) converts free-text impressions to structured, itemized findings. Examples of both outputs are shown. **b**, Raw CT/MRI volumes are first resampled to 1 × 1 × 4 mm (4 mm along the native acquisition axis) to reflect conventional clinical slice spacing. MRI intensities are clipped to 0.5-99.5th percentile, and CT volumes are windowed into brain, blood, and bone using standard Hounsfield windows. All volumes are quantized to 8-bit (CT blood/bone further reduced to 4-bit) to reduce footprint. Modality-specific foreground masks remove air only (no skull-stripping). The resulting dataset contains 11.8 billion 3D 4 × 16 × 16 patches (approximately 9 TB). **c**, Example CT/MRI volumes after background removal and tokenization. For Vol-JEPA training, volumes are partitioned into context-target pairs. Mask placements and lengths are randomized to cover diverse spatial configurations. **d**, In Vol-JEPA, a student volumetric transformer first encodes the context, after which a predictor combines these latents with the masked target positions to predict target latents. A teacher encoder (an EMA of the student) processes the full volume to produce target latents for the masked region. The objective minimizes the smooth L1 distance between the predicted and teacher target latents. Only the student and predictor receive gradients, and ultimately the student is used for downstream inference. **e**, To compare fairly across architectures, we use a unified study-level attentive probing strategy across all available tokens. 2D models (for example, DINOv3 and BiomedCLIP) encode each slice, with classification, register, and patch tokens from all slices fed to the study-level probe. 3D volume-level models (for example, NeuroVFM and NeuroMAE) encode each volume, with all tokens pooled jointly. 3D study-level models (for example, HLIP) natively output study classification tokens, but for parity we use all available output tokens in the study-level probe. **f**, We used NeuroVFM to condition a multimodal LLM in a LLaVA-1.5 style to generate preliminary neuroimaging findings. First, each volume in a study is encoded with frozen NeuroVFM. The resulting tokens are condensed by a per-volume Perceiver resampler to 64 latents. These then pass through the LLM connector (two-layer MLP) and are fed as vision tokens to a decoder-only LLM (for example, Qwen3-14B). During finetuning, the clinical indication is given as text input and the model is trained to generate the itemized findings from a. An example indication and generated findings are shown. **g**, Illustrative deployment of NeuroVFM within a secure hospital environment. Each time a patient undergoes neuroimaging, an internal function call applies NeuroVFM to generate preliminary findings. NeuroVFM and downstream on-premises modules act as perception models that ingest private inputs (for example, full 3D imaging and clinical indication) and produce de-identified structured findings, which can then be provided to an external reasoning model to support tasks such as triage, referral, or care pathway recommendations. Crucially, only structured findings are transmitted (no images, free-text reports, nor PHI), thereby reducing privacy risk while enabling use of state-of-the-art reasoning models.

**Extended Data Fig. 2:**
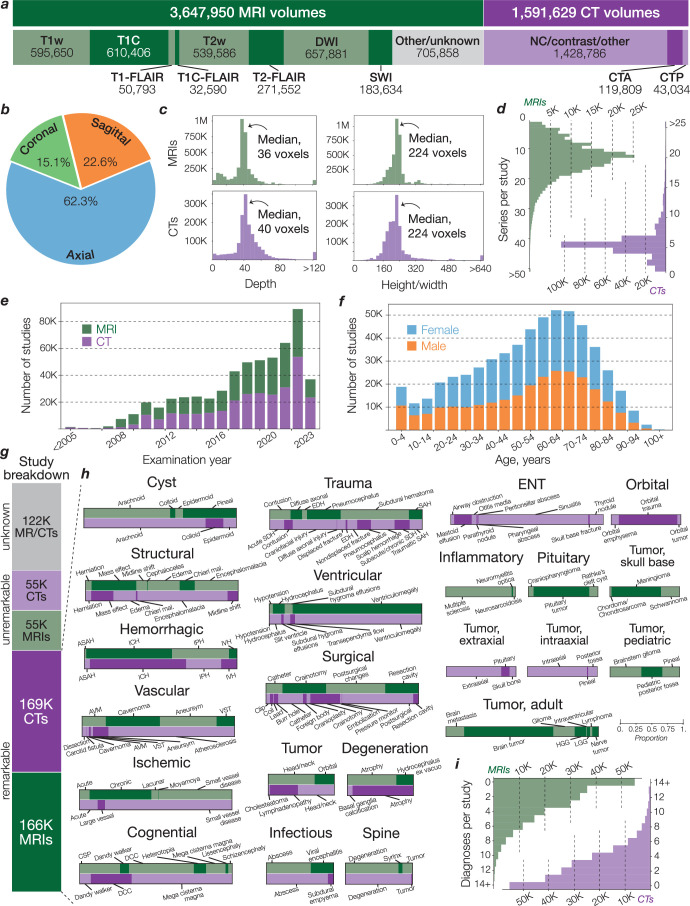
Overview of UM-NeuroImages. Descriptive statistics for the retrospective training corpus used for NeuroVFM. **a**, Volume counts by modality and sequence family. Sequence families were inferred from the DICOM ‘Series Description’ via keyword matching. Counts are therefore approximate and may not perfectly reflect acquisition parameters. **b**, Distribution of volume orientation (axial, sagittal, coronal). **c**, Voxel-space distributions by modality: depth (slices) and in-plane dimension (height/width). Medians are indicated (MRI depth, 36 slices; CT depth, 40 slices; in-plane size for both, 224 voxels). **d**, Distribution of series per study by modality, showing a higher number of series in MRI (mode, 12) relative to CT (mode, 5). **e**, Study counts by calendar year (2005-2023) for MRI and CT. June 1, 2023 marks the cut-off for inclusion in the training set. **f**, Study counts by patient age (5-year bins) and sex. **g**, Breakdown of studies by label status: remarkable (at least 1 positive diagnosis), unremarkable (no reported abnormality), and unknown (no paired report). Totals shown correspond to 168,579 CT and 165,780 MRI remarkable studies; 55,444 CT and 55,051 MRI unremarkable studies; and 122,061 studies with no retrieved report used for Vol-JEPA self-supervised pretraining. **h**, Per-category prevalence of diagnoses (82 CT labels, 74 MRI labels). The aim was to design a clinically relevant ontology of diagnostic classes to evaluate our representation models. We refer to this benchmark as UM-NeuroBench. Abbreviations: Hemorrhagic - ASAH, aneurysmal subarachnoid hemorrhage; ICH, intracranial hemorrhage; IPH, intraparenchymal hemorrhage; IVH, intraventricular hemorrhage. Vascular - AVM, arteriovenous malformation; VST, venous sinus thrombosis. Congenital - CSP, cavum septum pellucidum; DCC, dysgenesis of the corpus callosum. Trauma - EDH, epidural hematoma; SAH, subarachnoid hemorrhage; SDH, subdural hematoma. Tumor, adult - HGG, high-grade glioma; LGG, low-grade glioma. **i**, Number of positive diagnoses per study by modality. The long-tailed, multi-label distribution underscores our label breadth and enables training strong multi-diagnostic classifiers.

To train NeuroVFM, we assembled UM-NeuroImages, a multicenter, multimodal dataset comprising 566,915 CT and MRI studies (5.24 million three-dimensional (3D) volumes) of the brain, head, neck, face and orbits from over two decades of routine clinical care at Michigan Medicine (Fig. 1 and Extended Data Fig. 1). We defined a diagnostic ontology of 74 MRI and 82 CT diagnoses spanning neoplastic, traumatic, infectious, inflammatory and other major pathologic categories. Diagnostic labels were automatically assigned from radiology reports using a validated large language model (LLM) pipeline^4^, with a subset verified by expert neuroradiologists (Supplementary Table 1). These labels were not used for self-supervised pretraining and were used solely to train and evaluate supervised diagnostic heads. Detailed dataset characteristics, including sequence types, image resolutions and diagnosis distributions, are provided in Extended Data Fig. 2.

**Extended Data Fig. 3:**
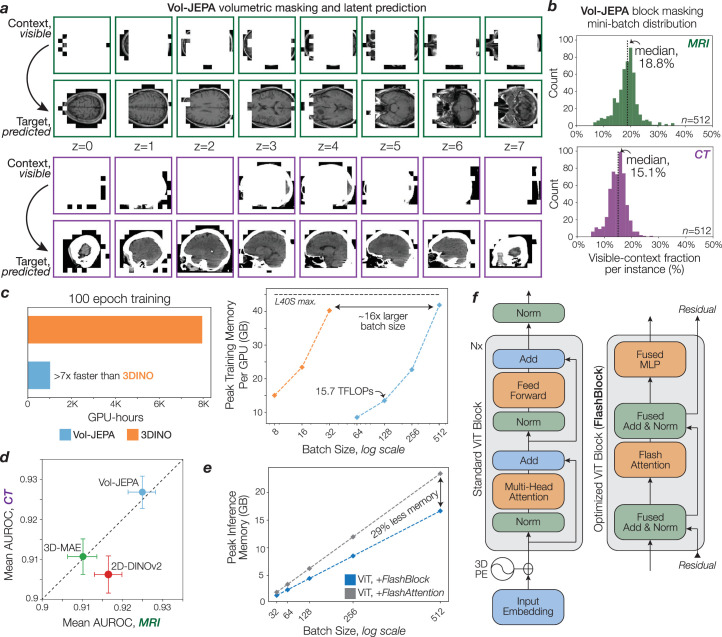
Learning with Vol-JEPA. **a**, For each Vol-JEPA training instance, we generate context-target pairs by sampling (1) one or more large blocks whose union forms the masked target, or (2) a single small block that serves as the visible context. These blocks span all three axes, ensuring the encoder sees a diverse set of spatial volumes. Examples are shown for MRI and CT volumes across eight axial slices (z=0-7). **b**, Mini-batch histograms (n=512) show the per-instance visible-context fraction (MRI median, 18.8%; CT median, 15.1%). CT uses slightly smaller contexts because out-of-body voxels are reliably background. Since MRI foregrounds often include air due to lack of explicit skull stripping, we keep a slightly larger visible fraction to preserve anatomical signal. The range of fractions encourages the encoder to model shared neuroanatomy rather than background shortcuts. **c**, Robust volumetric self-supervised learning is typically expensive due to heavy augmentations and large forward passes. Left, a 3D-DINO-style baseline using MONAI 3D augmentations require > 7 × GPU-hours than Vol-JEPA to train for 100 epochs. Right, peak training memory per GPU versus. batch size (log scale) shows Vol-JEPA encoding 16 × larger batches for any given memory below the L40S limit. At batch size 128, throughput is approximately 15.7 TFLOPs/GPU. **d**, We trained a compute-matched voxel reconstruction baseline, termed NeuroMAE, (85% random masking) and DINOv2 on 2D images (UM-NeuroImages training corpus, with 209M unique slices). Using the unified study-level attentive probing protocol (Extended Data Fig. 1e), Vol-JEPA attains the highest mean AUROC on UM-NeuroBench for MRI and CT, indicating advantages of masked volumetric latent prediction over decoding-centric (MAE) and 2D slice-only (DINOv2) approaches. Results are mean ± 95% boostrapped CIs (10,000 replicates). **e**, We replaced the standard pre-norm ViT block (with FlashAttention-2) with a FlashAttention-2 block with operator fusion (Tri Dao, 2023). Benchmarking demonstrated 29% lower peak inference memory used across batch sizes for ViT+FlashBlock versus a standard ViT+FlashAttention stack. **f**, Schematic of the FlashBlock architecture versus a conventional ViT block with FlashAttention.

JEPAs are self-supervised learning methods that, given a context region within a data sample, predict a non-overlapping target region in a learned latent space. JEPAs have achieved state-of-the-art performance on image and video data but have not been applied to volumetric medical images^5–7^. We adapted this framework for volumetric neuroimaging (Vol-JEPA), designing a neuroanatomy-informed masking strategy that exploits the shared spatial structure in neuroimages (Fig. 1b and Extended Data Fig. 3a). We hypothesized that this objective would require the encoder to learn representations invariant to imaging protocol and equivariant to neuroanatomy and pathology. Because Vol-JEPA learns through latent prediction, it does not require voxel-level augmentations, negative pairs, generative decoders or paired radiology reports, enabling efficient scaling to large, uncurated clinical datasets. Vol-JEPA outperformed alternative self-supervised strategies, including contrastive and reconstruction-based objectives, on downstream diagnostic tasks (Extended Data Fig. 3c).

### Testing NeuroVFM on diverse neuroimaging tasks

To compare health system learning with existing approaches, we curated a temporally held-out diagnostic cohort comprising all consecutively evaluated patients who underwent a CT or MRI scan of the head or neck between 1 June 2023 and 31 May 2024, without exclusion, yielding more than 21,000 CT and 29,000 MRI studies (Supplementary Table 2). We compared NeuroVFM against five baselines spanning report-supervised health system pretraining (HLIP^8^ and Prima^4^), voxel reconstruction self-supervision on the same health system data (NeuroMAE), internet-scale self-supervised learning (DINOv3 (ref. ^9^)) and biomedical vision-language pretraining (BiomedCLIP^10^). For each model, we standardized evaluation by training identical study-level attentive probes on frozen encoder embeddings.

**Fig. 2:**
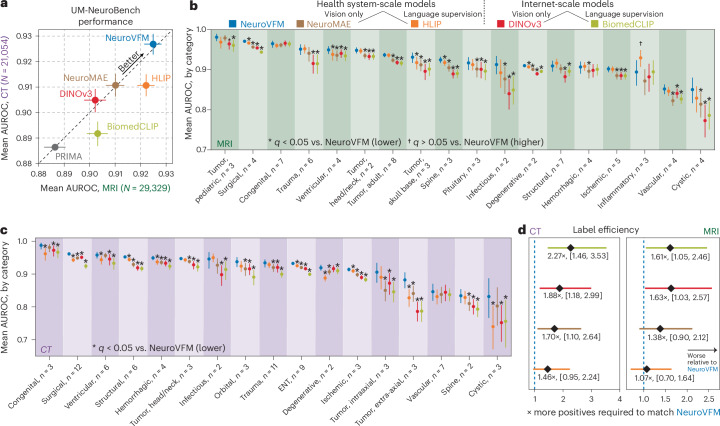
NeuroVFM results. **a**, NeuroVFM performance over 82 CT and 74 MRI diagnostic tasks, compared to both health system-scale (HLIP, PRIMA) and internet-scale (DINOv3, BiomedCLIP) models. NeuroVFM outperforms models trained with language supervision and those trained on public internet data. PRIMA was trained and evaluated only on MRI scans. Results are mean ± 95% CI. **b**,**c**, Performance across diagnostic ontologies, such as traumatic, congenital and ischemic lesions, is shown for both CT (**c**) and MRI (**b**). NeuroVFM consistently outperforms other baselines. Results are mean ± 95% CI. Significance was computed using paired bootstrap, corrected for multiple hypotheses using Benjamini−Hochberg with a false discovery rate of 0.05. *n* refers to the number of tasks per ontology. ENT stands for ear, nose and throat. **d**, We discovered an empirical log-linear scaling relationship between the number of positive training examples and model performance. This relationship held across at least four orders of magnitude, imaging modalities and models (Extended Data Figs. 4 and 5). We plot label equivalence factors for baselines relative to NeuroVFM, which requires fewer training positives to reach the same performance. Results are mean ± 95% CI.

On the primary endpoint (macro-averaged area under the receiver operating characteristic (AUROC) across all 156 diagnostic tasks), NeuroVFM achieved 92.68 (95% confidence interval (CI) 92.27−93.08) on CT and 92.49 (95% CI 92.14−92.82) on MRI (Fig. 2a). Because PRIMA, HLIP, NeuroMAE and NeuroVFM were all pretrained on UM-NeuroImages, these comparisons control for data source, and differences are attributed to pretraining objective and model design. NeuroVFM outperformed both report supervision (HLIP: +0.98, 95% CI 0.76−1.20; PRIMA: +3.87, 95% CI 3.53−4.21) and voxel reconstruction (NeuroMAE: +1.55, 95% CI 1.35−1.75). The largest margins were over internet-scale baselines (DINOv3: +2.24, 95% CI 1.97−2.50; BiomedCLIP: +2.88, 95% CI 2.62−3.15), where differences in both objective and pretraining data compound. For individual pathology categories, NeuroVFM exceeded baselines broadly after Benjamini−Hochberg correction (*q* < 0.05; Fig. 2b,c). NeuroVFM significantly outperformed DINOv3, BiomedCLIP and NeuroMAE in at least 12 of 17 CT categories and 14 of 18 MRI categories. Against HLIP, NeuroVFM won 10 of 17 CT categories with no losses, but only five of 18 MRI categories, with HLIP winning the inflammatory category and the rest being non-significant. NeuroVFM also demonstrated better label efficiency, requiring fewer labeled positive examples than each baseline (31.5–55.9% fewer CT scans; 6.5–37.9% fewer MRI scans) to reach equivalent performance (Fig. 2d). Per-task and per-category results are in Supplementary Tables 3 and 4, respectively.

**Extended Data Fig. 4:**
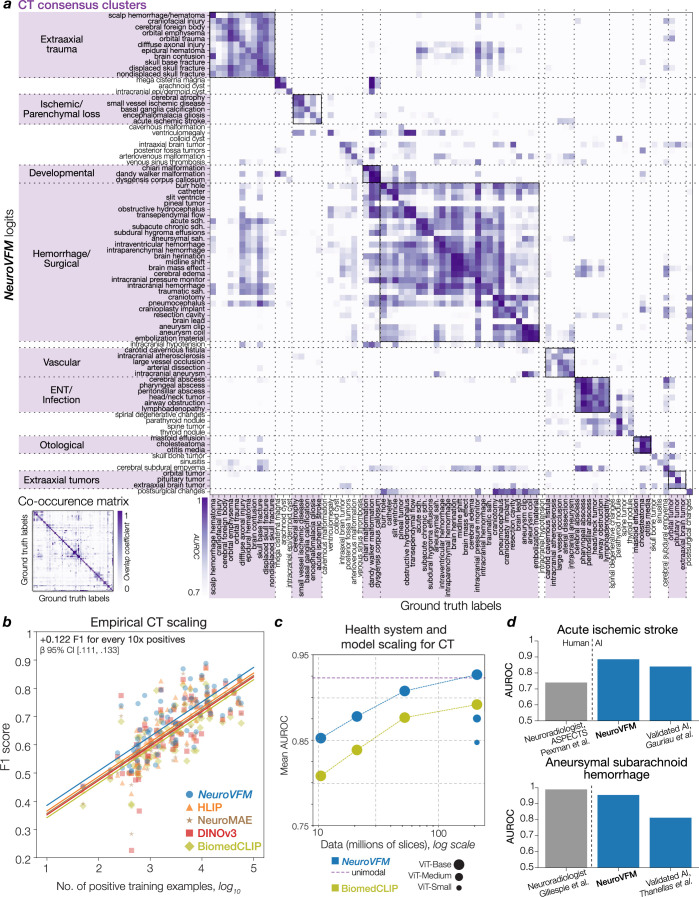
Extended CT diagnostic results. CT diagnostic evaluation comprises 82 diagnoses defined on UM-NeuroImages. All encoders were frozen and evaluated using the same study-level attentive probing strategy (Extended Data Fig. 1e). **a**, Logit-label performance matrix for the NeuroVFM CT classifier, where each cell is AUROC of logit*i* against ground-truth label_*j*_ (main diagonal shows per-diagnosis AUROC). Rows and columns are reorganized by hierarchical consensus clustering (bootstrapped similarity, average linkage). Lower left, reference co-occurrence matrix computed from ground truth using min-normalization. **b**, We fit a shared slope across NeuroVFM and three baselines (BiomedCLIP, DINOv3, and HLIP) via ANCOVA, relating calibrated F1 score to log10 of the number of positive training instances. Intercept shifts yield data-equivalence factors (how many more positives a baseline required to match NeuroVFM at a fixed F1), shown in Fig. 2d. **c**, Data and model size ablation results plotted for NeuroVFM on the CT subset of UM-NeuroImages. Performance increases steadily along both axes, suggesting continued improvement with more data and larger models. BiomedCLIP is shown as a baseline comparator, demonstrating that NeuroVFM’s performance is not just a function of more data. **d**, We compared NeuroVFM results on two representative CT detection tasks (top, acute ischemic stroke; bottom, aneurysmal subarachnoid hemorrhage) to published studies. NeuroVFM achieves AUROC comparable to expert neuroradiologists ^51,52^ [refs. ^52,53^] and to validated AI systems ^53,54^ [refs. ^54,55^]. For prior studies, AUROC is approximated as the mean of the reported sensitivity and specificity.

**Extended Data Fig. 5:**
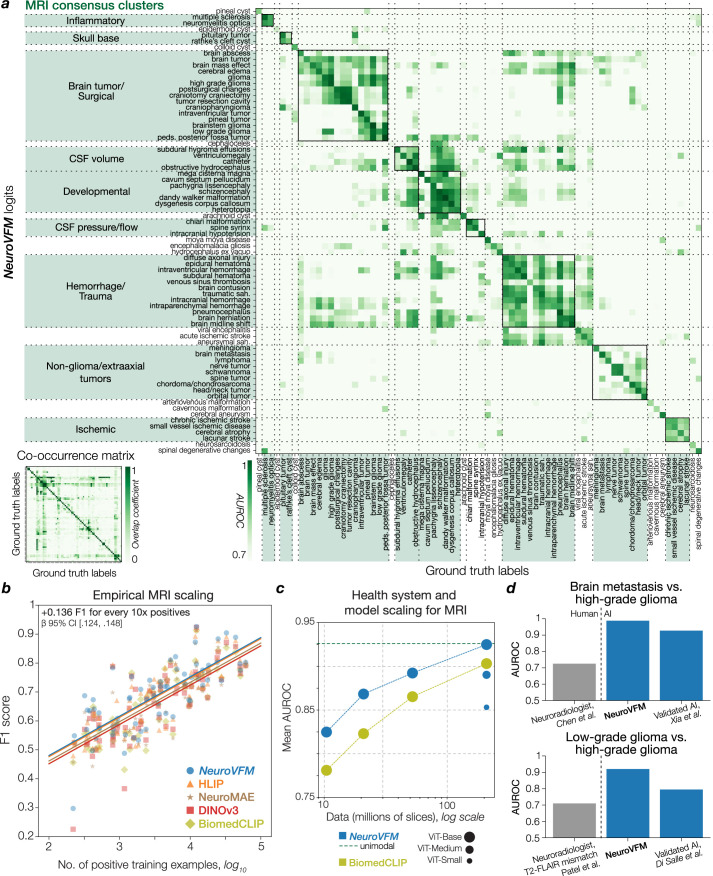
Extended MRI diagnostic results. MRI diagnostic evaluation comprises 74 diagnoses defined on UM-NeuroImages. All encoders were frozen and evaluated using the same study-level attentive probing strategy (Extended Data Fig. 1e). **a**, Logit-label performance matrix for the NeuroVFM MRI classifier, where each cell is AUROC of logit*i* against ground-truth label_*j*_ (main diagonal shows per-diagnosis AUROC). Rows and columns are reorganized by hierarchical consensus clustering (bootstrapped similarity, average linkage). Lower left, reference co-occurrence matrix computed from ground truth using min-normalization. **b**, We fit a shared slope across NeuroVFM and three baselines (BiomedCLIP, DINOv3, and HLIP) via ANCOVA, relating calibrated F1 score to log10 of the number of positive training instances. Intercept shifts yield data-equivalence factors (how many more positives a baseline requires to match NeuroVFM at a fixed F1), shown with 95% CIs. **c**, Data and model size ablation results plotted for NeuroVFM on the MRI subset of UM-NeuroImages. Performance increases steadily along both axes, suggesting continued improvement with more data and larger models. BiomedCLIP is shown as a baseline comparator, demonstrating that NeuroVFM’s performance is not just a function of more data. **d**, We compared NeuroVFM results on two representative MRI differentiation tasks (top, brain metastasis versus high-grade glioma; bottom, low- versus. high-grade glioma) to published studies. For NeuroVFM, the score was the pairwise logit margin (logit1-logit2), and AUROC was computed on this margin. NeuroVFM achieves AUROC comparable to expert neuroradiologists ^55,56^ [refs. ^56,57^] and to validated AI systems ^57,58^ [refs. ^58,59^]. For prior studies that only report sensitivity and specificity, we approximate the AUROC as the average.

**Extended Data Fig. 6:**
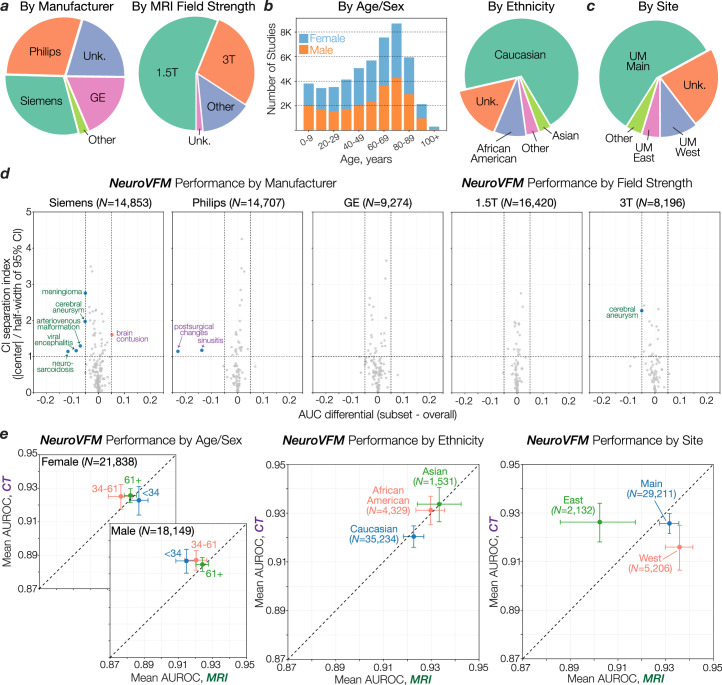
Subgroup and health system analysis. Descriptive statistics for the testing corpus and robustness of NeuroVFM across common sources of health system variation. **a**, Composition by scanner manufacturer and MRI field strength. **b**, Composition by age/sex and ethnicity. **c**, Composition by acquisition site within the health system. We limited the analysis to the three most common sites: University of Michigan (UM) Main, West, and East. **d**, Per-diagnosis robustness checks by (left) manufacturer and (right) MRI field strength. For each eligible cell (subgroup × diagnosis; requires at least 10 positives and negatives present), we compute the AUC differential relative to the full test set (Δ_*g*_ = AUC_*g*_ − AUC_all_) using 10,000 paired, study-level bootstrap resamples to form percentile 95% CIs. Points show Δ_*g*_, and the vertical dashed lines mark an a priori equivalence band of ± 0.05 AUROC. The y-axis summarizes CI separation index, $S=\frac{{\Delta }_{g}}{{\rm{half}}}-{{\rm{width}}}_{95 \% }$, where *S* > 1 indicates the 95% CI excludes 0 (that is, a significant deviation). Cells with 95% CIs entirely within ± 0.05 AUROC were interpreted as showing no material deviation. Most diagnoses remain within the equivalence band, with outliers annotated (CT, purple; MRI, green). **e**, Mean AUROC ( ± 95% CIs) for MRI versus CT stratified by age/sex, ethnicity, and site. No material differences present for age/sex and ethnicity. At the site level, MRI performance is lower at the East site, while CT is comparable across all.

Performance scaled with both pretraining data and encoder capacity, improving by +7−10 AUROC points from 5% to 100% of UM-NeuroImages on both modalities (Extended Data Figs. 4c and 5c). These trends showed no evidence of saturation, suggesting continued benefit from additional health system data. Multimodal pretraining on both CT and MRI scans was non-inferior to unimodal models, indicating that learning a shared latent space did not degrade within-modality performance. NeuroVFM also maintained performance across MRI manufacturers, magnetic field strengths, demographic subgroups and medical centers (Extended Data Fig. 6).

**Extended Data Fig. 7:**
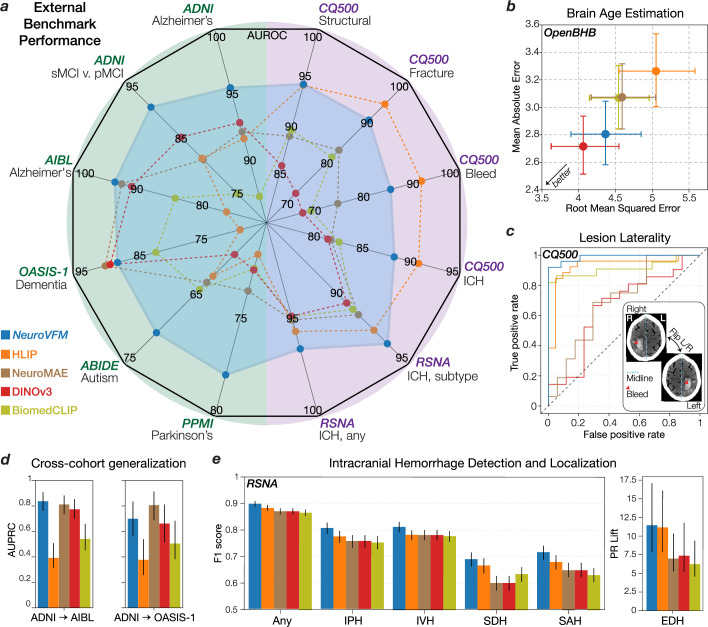
Open benchmark performance. All encoders were frozen and evaluated using the same study-level attentive probe (Extended Data Fig. 1e). Error bars show 95% bootstrapped CIs (10,000 replicates). **a**, Radar plot depicting AUROC performance for all baselines across many open neuroimaging benchmarks. Each ring along a spoke depicts a +5% increase in AUROC. NeuroVFM consistently demonstrates strong performance across all tasks, with the exception of CQ500, where the limited training data favors a language-supervised encoder like HLIP. Full results can be found in Supplementary Data Table 5. **b**, Brain age estimation results on the OpenBHB dataset (T1 MRIs). Scatter of RMSE (x-axis) versus MAE (y-axis) per model shown. NeuroVFM is competitive (MAE, 2.805 years), second to BiomedCLIP. Strong 2D baselines likely benefit from dense slice-level features available to the probe. **c**, Correct laterality assignment is clinically important yet rarely tested. The CQ500 dataset provides expert ‘Bleed-Left/Bleed-Right’ labels, allowing a targeted laterality check given correct bleed detection. For each model, among studies it correctly flags as bleed, we horizontally flip the volume and measure changes in both the Bleed-Left and Bleed-Right logits (that is, ${\Delta }_{L}={{\rm{logit}}}_{L}^{{\rm{flipped}}}-{{\rm{logit}}}_{L}$, ${\Delta }_{R}={{\rm{logit}}}_{R}^{{\rm{flipped}}}-{{\rm{logit}}}_{R}$). We summarize performance as AUROC over Δ_*L*_ and Δ_*R*_ against the ground-truth side. NeuroVFM is near perfect, representative of strong anatomical encoding. **d**, We used three Alzheimer’s/dementia cohorts to test whether features generalize beyond a single study: ADNI (training, with a held-out test set) and two independent test sets (AIBL and OASIS-1). ADNI to AIBL reflects classic domain shift, whereas ADNI to OASIS-1 probes robustness as OASIS-1 provides a dementia severity score (CDR 0 versus ≥1). Training on ADNI and testing on AIBL/OASIS-1 yields strong AUPRC for NeuroVFM (NeuroMAE and DINOv3 comparable). These results indicate the learned self-supervised features capture disease-relevant information rather than site-specific cues. e, Intracranial hemorrhage detection and localization results on the 2019 RSNA-ICH dataset (non-contrast CT). Left, F1 for ICH detection and each subtype (IPH, IVH, SDH, SAH) shown. Right, PR lift for EDH (AUPRC/prevalence; EDH, 1.88%). NeuroVFM matches or exceeds other baselines across all tasks.

On public neuroimaging benchmarks spanning brain age estimation, neurodegenerative and neurodevelopmental disorders and traumatic brain injury (Extended Data Fig. 7), NeuroVFM achieved the strongest performance on tasks whose labels are not directly encoded in radiology reports. Using classifiers trained on the Alzheimerʼs Disease Neuroimaging Initiative (ADNI) cognitively normal versus Alzheimerʼs disease task, NeuroVFM generalized strongly to external cohorts, achieving AUROCs of 93.49 (95% CI 90.03−96.40) on AIBL and 88.09 (95% CI 80.47−94.24) on OASIS-1 compared to 76.18 (95% CI 70.16−81.77) and 69.08 (95% CI 58.84−78.56) for HLIP, respectively. HLIP outperformed NeuroVFM on most CQ500 tasks, a small CT benchmark with label definitions closely aligned to report descriptions. This advantage diminished on the larger RSNA-ICH benchmark, where NeuroVFM exceeded HLIP. Together, these results provide evidence that report-supervised models can perform well within specific diagnoses, whereas NeuroVFM representations generalize better as task diversity increases. Full results across all evaluated public benchmarks are in Supplementary Table 5.

### NeuroVFM jointly encodes spatial and semantic information

**Fig. 3:**
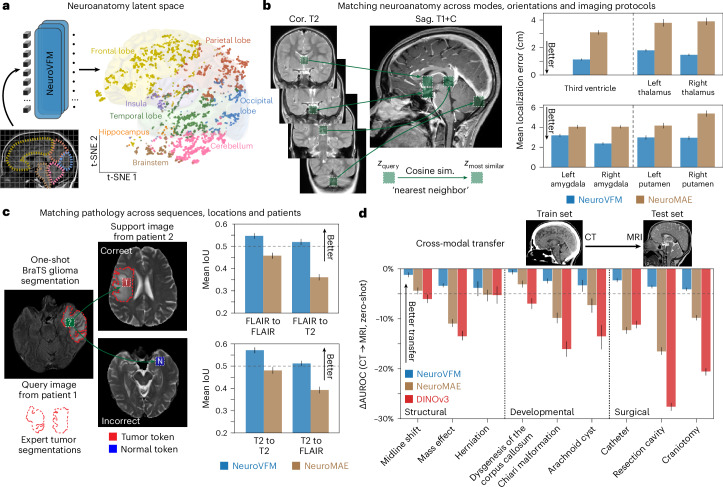
NeuroVFM learned a joint neuroimaging latent space. **a**, Patch embeddings from a T1-weighted MRI sequence plotted using t-SNE and colored by neuroanatomical region from SynthSeg segmentation^28^. NeuroVFM representations cluster by anatomy without explicit supervision during pretraining. Language-supervised baselines were not evaluated here because their training objectives do not explicitly learn dense patch-level representations, and cross-modal alignment is an expected result of CLIP. **b**, Cross-protocol anatomical matching accuracy across seven landmarks compared to NeuroMAE, a voxel reconstruction baseline. Results are mean ± two-way cluster-robust standard error. **c**, One-shot tumor retrieval over BraTS21 sequences using paired glioma segmentation masks^12^. Given a query patch, the nearest neighbor in a support image transfers its label. NeuroVFM achieves IoU greater than 0.5, even across sequence types. Results are mean ± two-way cluster-robust standard error. **d**, Zero-shot cross-modal diagnostic transfer performance, with CT-trained attentive classifier evaluated on MRI across nine shared diagnostic labels. NeuroVFM exhibits less than a 5-point AUROC drop, whereas other baselines degrade substantially, indicating that their representations do not generalize across modalities. Results are mean ± 95% CI. Horizontal dotted lines in **c** and **d** indicate reference benchmarks for interpretation. Cor., coronal; Sag., sagittal; sim., similarity; t-SNE, t-distributed stochastic neighbor embedding.

Self-supervised learning on large-scale natural image and video datasets can give rise to representations with rich spatial and semantic features^9,11^. Achieving similar results in medical foundation models remains a major challenge. NeuroVFM patch embeddings reveal spatially ordered neuroanatomic clusters, without needing segmentation or report supervision during pretraining (Fig. 3a). These results indicate that NeuroVFM has learned a neuroanatomic manifold that encodes semantic and spatial information. We quantified these properties through three evaluations of increasing complexity: anatomical matching across patients, pathology retrieval across patients and MRI sequences and zero-shot diagnostic transfer across patients and modalities.

**Extended Data Fig. 8:**
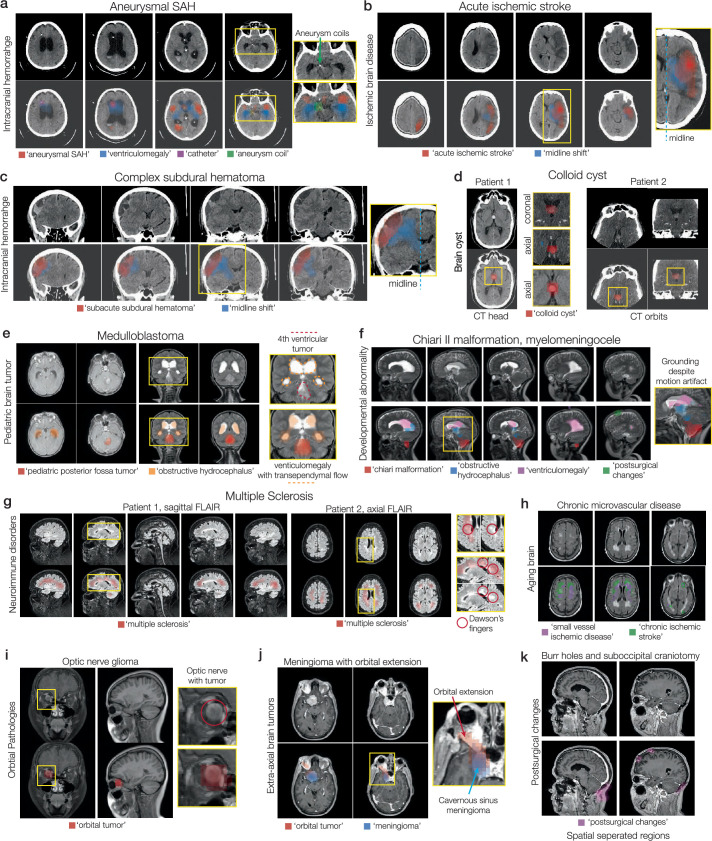
Grounded NeuroVFM diagnoses. A diverse set of illustrative examples that demonstrate NeuroVFM is a grounded visual foundation model. **a**, An example of aneurysmal subarachnoid hemorrhage, treated via endovascular coiling, with associated hydrocephalus and ventricular catheter. NeuroVFM can parse the head CT into diagnostic regions and associate correct diagnoses. **b**, A patient with an acute middle cerebral artery ischemic stroke and associated cerebral edema and midline shift. **c**, A patient with a complex, septated, mixed-density subdural hematoma also causing midline shift. Note that NeuroVFM identifies midline shift regardless of the underlying etiology. **d**, Two patients with classic third ventricle colloid cysts. NeuroVFM attends to the colloid cyst regardless of patient orientation or imaging protocol. **e**, An example of a pediatric posterior fossa tumor causing obstructive hydrocephalus. NeuroVFM performs well across age groups. **f**, Example of a Chiari II malformation with associated obstructive hydrocephalus, ventriculomegaly, and previous cerebrospinal shunt placement. **g**, Two patients with newly diagnosed multiple sclerosis. Both patients have classic periventricular demyelination, known as Dawson’s fingers, which NeuroVFM identifies on both sagittal and axial orientations. **h**, NeuroVFM can differentiate between multiple sclerosis and periventricular white matter changes due to chronic microvascular disease. An example of an (**i**) optic nerve glioma and a (**j**) cavernous sinus meningioma with intraorbital extension. NeuroVFM performance generalizes well to ophthalmologic conditions. **k**, NeuroVFM can associate spatially separated regions, for example, surgeries completed in different locations, with the same underlying diagnosis. NeuroVFM can identify postsurgical changes throughout the neuroimaging study.

To test whether NeuroVFM encodes shared neuroanatomy across imaging protocols, we performed nearest-neighbor patch matching of anatomical landmarks across image pairs differing in both sequence and orientation (for example, coronal T2 and sagittal T1 with contrast). Across seven landmarks, NeuroVFM achieved a 44.2% lower mean localization error compared to NeuroMAE (2.27 cm versus 4.07 cm), indicating that neuroanatomic information is invariant to acquisition parameters. This is consistent with the fine-grained spatial localization and grounded diagnosis achieved by NeuroVFM-based classifiers on both MRI and CT (Extended Data Fig. 8).

To determine whether this invariance extended to pathology, we designed a one-shot tumor retrieval task using BraTS21 (ref. ^12^) segmentation masks: given a query patch, we retrieve its nearest-neighbor patch in a support image and transfer its label (Fig. 3c). Pairs were selected such that tumors occupied different anatomical locations, and performance was evaluated across MRI sequences (for example, fluid-attenuated inversion recovery (FLAIR) to T2). NeuroVFM achieved intersection over union (IoU) exceeding 50% regardless of transfer direction, whereas NeuroMAE performed worse within the same sequence and degraded further across sequences. To test whether this generalizes to study-level diagnosis, we evaluated CT-trained attentive classifiers on MRI, measuring the AUROC drop relative to within-modality performance (Fig. 3d). Across nine shared diagnostic tasks, NeuroVFM exhibited less than a 5-point drop in AUROC, outperforming NeuroMAE and DINOv3 on eight of nine tasks. These results demonstrate that NeuroVFM has learned a modality-agnostic representation of neuroanatomy and disease. These properties do not emerge from alternative training objectives applied to the same data, indicating that they arise at the intersection of volumetric latent prediction and large-scale neuroimaging datasets.

### NeuroVFM enables preliminary report generation

**Fig. 4:**
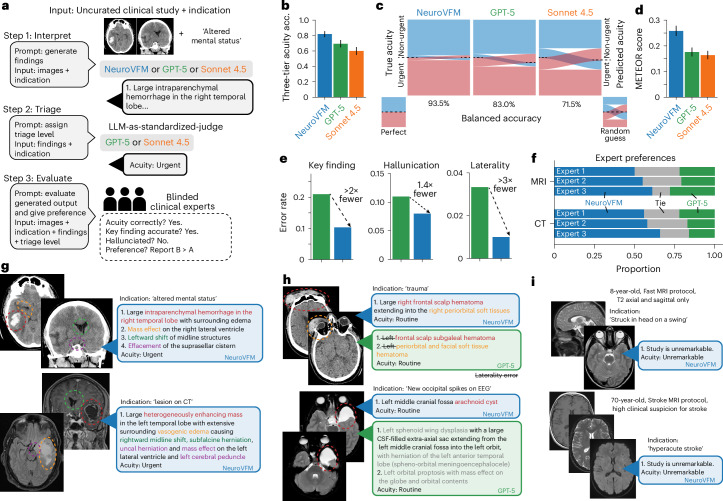
Preliminary report generation results. **a**, Overview of the study design and workflow for report generation and acuity assessment. Step 1: a generative multimodal model, such as NeuroVFM-LLaVA or GPT-5, is prompted to generate image key findings. Step 2: an LLM, GPT-5 or Claude Sonnet 4.5, is then used as a standardized judge to evaluate the study or acuity level based on the findings generated in step 1. Step 3: ‘Blinded’ clinical experts then evaluate whether the study was stratified correctly, the quality of the generated reports and which report they prefer. NeuroVFM outperformed the other frontier models on overall acuity performance (**b**), urgent findings generation (**c**) and all natural language processing metrics (**d** and Extended Data Fig. 9). Results are mean ± 95% CI. **e**, Blinded clinical experts noted that NeuroVFM was more likely to generate correct key findings, less likely to hallucinate and much less likely to make a laterality error compared to GPT-5. **f**, Clinical experts also preferred NeuroVFM reports more than 2:1 over GPT-5 reports. **g**−**i**, Illustrative examples of urgent (**g**), routine (**h**) and unremarkable (**i**) neuroimaging studies in the expert-annotated testing set. Results are mean ± 95% CI. acc., accuracy; CSF, cerebrospinal fluid.

Generating accurate radiology findings from the raw clinical imaging stream is a prerequisite for enabling real-time worklist prioritization, reducing reporting delays and extending expert-level interpretation to settings without subspecialty coverage^13,14^. Unlike curated benchmarks, report generation requires operating on the full diversity of studies encountered in routine practice: motion artifact, variable protocols, incidental findings and the long tail of rare pathology. We tested whether NeuroVFM could support this task by training a simple generative model to produce structured findings from ‘uncurated clinical neuroimaging studies’ (Fig. 4a).

**Extended Data Fig. 9:**
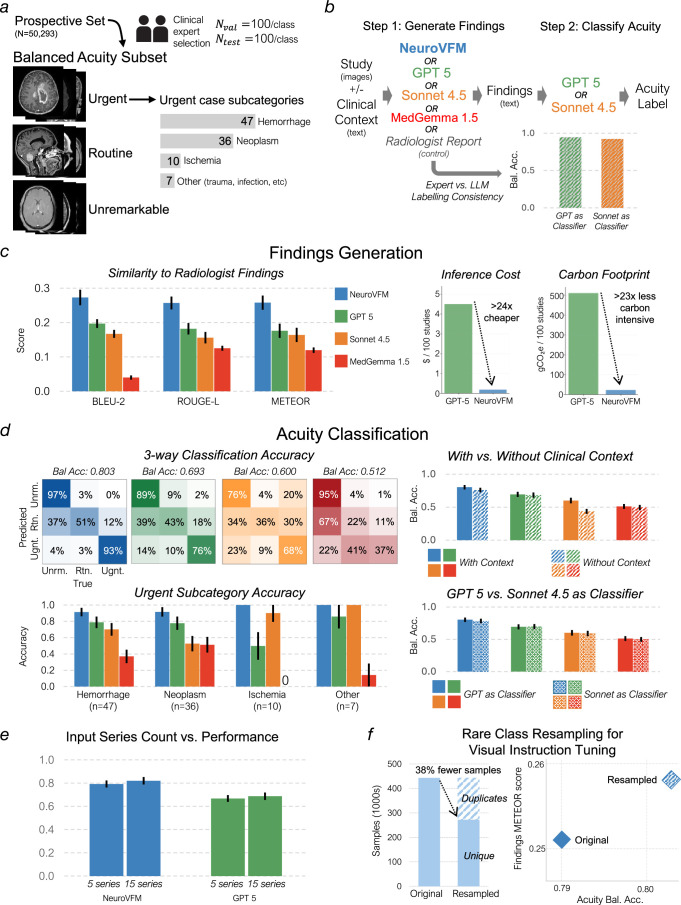
Generative evaluation. Error bars are 95% bootstrapped CIs (10,000 replicates). **a**, Composition of the UM-NeuroImages-Acuity evaluation dataset. The set consists of 600 studies (300 validation, 300 holdout) derived from our test set. This set was hand-selected by neuroimaging experts to be balanced across three acuity classes (‘Unremarkable’, ‘Routine’, ‘Urgent’) and two modalities (MRI, CT). **b**, Schematic of the two-stage evaluation pipeline for neuroimaging report generation and acuity assessment. Step 1 (Generate Findings): 3D studies (for NeuroVFM) or converted 2D slices (for GPT-5, Claude Sonnet 4.5, and MedGemma 1.5) are provided to each model with a one-sentence clinical indication to generate key findings. Step 2 (Classify Acuity): The generated text is passed to an LLM (GPT-5 or Claude Sonnet 4.5) to assess acuity. The pipeline was validated by passing ground-truth radiologist findings to the LLMs with the same classification prompt, achieving > 95% accuracy in replicating the expert-designated acuity labels. **c**, Generated report quality and inference cost analysis. Left: Bar plots comparing NeuroVFM, GPT-5, Claude Sonnet 4.5, and MedGemma 1.5 on traditional NLP metrics (BLEU-2, ROUGE-L, METEOR) against ground-truth summarized reports. Right: Post-hoc analysis of inference efficiency, comparing dollar cost (USD) and carbon footprint (gCO2e) per 100 studies. NeuroVFM estimates assume inference on a single NVIDIA L40S GPU (AWS G6e instance) with a 3s/study inference time and southeast Michigan power grid intensity (0.5869 kgCO2e/kWh). GPT-5 estimates use October 2025 batch API pricing and carbon estimates from Jegham et al. (medium reasoning, > 10k input/ > 1k output tokens)^59^. **d**, Acuity assessment performance and ablation studies. Top Left: 3-tier acuity accuracy for NeuroVFM, GPT-5, Claude Sonnet 4.5, and MedGemma 1.5 evaluated by the ‘LLM-as-a-judge’ pipeline. Bottom Left: Acuity accuracy stratified by key acute pathologies (for example, hemorrhage, aggressive mass, ischemia). Top Right: Ablation analysis on the impact of withholding the 1-sentence clinical indication associated with each imaging study from model input. Bottom Right: Ablation analysis on the robustness of acuity accuracy when using different ‘LLM-as-a-judge’ models (GPT-5 versus Claude Sonnet 4.5). **e**, Ablation analysis on the impact of input series count on triage performance. For both NeuroVFM and GPT-5 there is no significant difference in acuity performance when restricting to 5 input series vs. 15 series. **f**, Comparison of NeuroVFM performance between the final model (trained on a dataset resampled based on diagnostic class rarity) and a baseline model trained on the original, imbalanced dataset, demonstrating the performance gains from data curation.

We paired the frozen NeuroVFM encoder with Qwen3-14B via LLaVA-1.5-style visual instruction tuning^15,16^ and fine-tuned the language model to generate structured key findings from UM-NeuroImages studies. Generated findings were then passed to a fixed reasoning model that assessed acuity (unremarkable, routine or urgent), providing a model-agnostic evaluation of visual perception quality (Supplementary Fig. 1). We validated this protocol by confirming that frontier reasoning models achieved greater than 96% accuracy when given the ground truth radiology findings, establishing that acuity differences between models reflect perception quality rather than reasoning ability (Extended Data Fig. 9b). Multimodal frontier models (GPT-5 and Claude Sonnet 4.5) with ‘provider-imposed input constraints’ were the strongest available comparators, as existing open-source medical vision-language models (for example, MedGemma) failed to produce clinically usable findings (Extended Data Fig. 9 and Supplementary Table 6).

All models were tested on 300 expert-verified CT and MRI studies balanced across modality and acuity level. NeuroVFM-LLaVA outperformed both baselines on three-tier acuity accuracy (GPT-5: +11.0, 95% CI 5.7−16.2; Claude Sonnet 4.5 +20.3, 95% CI 14.3−26.2) and detection of urgent findings (GPT-5: +10.5, 95% CI 5.9−15.3; Claude Sonnet 4.5: +21.9, 95% CI 16.1−27.7) (Fig. 4b,c). Generated findings achieved higher scores on all natural language processing metrics computed against ground truth reports, including METEOR and ROUGE (Fig. 4d and Extended Data Fig. 9). In blinded expert evaluation, the key finding error rate of NeuroVFM-generated reports was approximately half that of GPT-5 (10% versus 20%), with fewer hallucinations and laterality errors (Fig. 4e). Three blinded clinical experts (R.J., A.-K.M. and T.H.) preferred NeuroVFM-generated reports over GPT-5 more than two to one, with high interrater concordance (Fig. 4f; Fleissʼ *κ* = 0.718).

**Extended Data Fig. 10:**
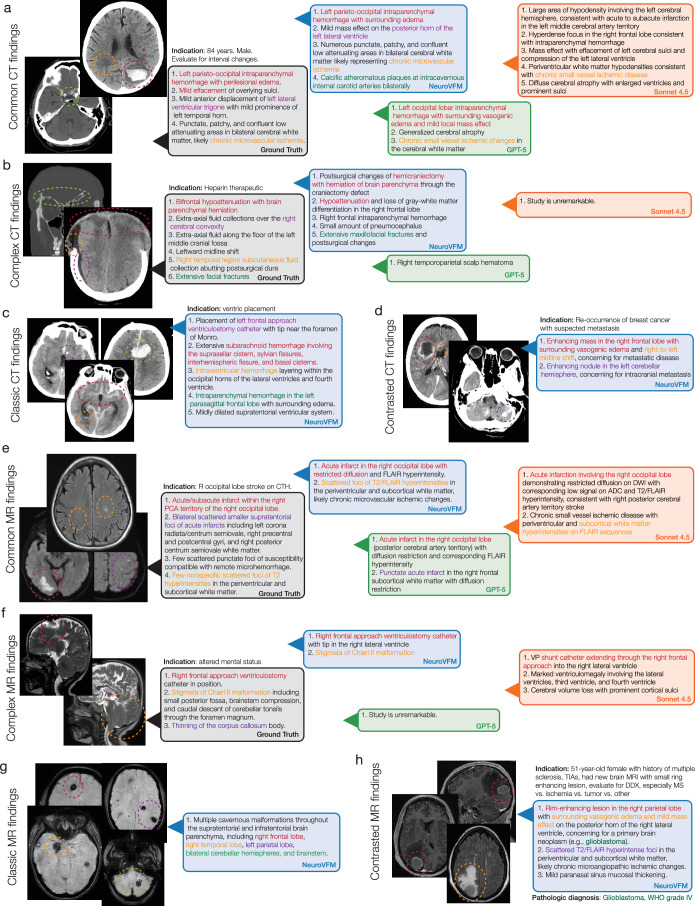
Generated reports. A diverse set of illustrative examples of generated reports. **a**, Example of a patient with a common lobar hemorrhage within the left parietal and occipital lobes. NeuroVFM and GPT-5 identified the hemorrhage and correctly localized it. **b**, A complex head CT of a patient who underwent a craniectomy after a traumatic brain injury. This example illustrates the challenge of interpreting real-world clinical neuroimaging studies that do not fit neatly into singular diagnoses, which is often observed in standardized research datasets. NeuroVFM was able to recognize the postsurgical and post-traumatic changes, while both GPT-5 and Claude Sonnet 4.5 were not able to interpret this image correctly. **c**, Classic subarachnoid hemorrhage pattern due to a ruptured anterior communicating artery aneurysm. **d**, Contrasted CTs are much less common. NeuroVFM was able to both recognize that this was a contrasted CT and interpret that the findings were most consistent with metastatic brain disease. **e**, A common example of restricted diffusion due to an acute infarct located in the right posterior cerebral artery (PCA) distribution. **f**, A complex patient with Chiari II malformation and myelomeningocele. NeuroVFM was trained on pediatric MRIs with developmental abnormalities and is, therefore, able to correctly interpret this MRI. **g**, Classic appearance of cerebral cavernous malformations. **h**, A patient with a complex medical history and indication has a new brain lesion. NeuroVFM correctly identified this tumor as most likely a glioblastoma, confirmed on pathologic diagnosis.

Figure 4g−i shows representative NeuroVFM-generated reports across urgent, routine and unremarkable acuity levels. NeuroVFM accurately identified large mass lesions, including intraparenchymal hematomas and infiltrative tumors, and recognized associated findings such as midline shift, effacement of the basal cisterns and brain herniation to determine lesion severity. Generated report examples spanning the full range of pathologies encountered in clinical practice are shown in Extended Data Fig. 10. We included an error analysis and a clinical example of NeuroVFM-assisted diagnosis in Supplementary Figs. 2 and 3.

### Prospective feasibility study of report sufficiency for triage

We next asked if model-generated reports are sufficient to support accurate critical findings and triage decisions in a clinical neuroradiology workflow. In a silent, 1-week, prospective, health-system-wide feasibility study, we compared two report generation arms: a NeuroVFM-LLaVA arm and a GPT-5 arm. Over the consecutive 1-week prospective window (18−25 January 2026), we generated reports for all CT and MRI studies of the head, brain, face, neck and orbits performed in routine care across our health system (*n* = 1,155; 601 MRIs and 544 CTs). To isolate the effect of report quality from triage reasoning, both arms used the same two-stage workflow (Supplementary Fig. 2a). First, we used a consensus list of critical neuroradiology findings as defined by the American Society of Neuroradiology (Supplementary Fig. 2d)^17^. This list was provided to an LLM screening model and used to flag generated reports that could plausibly contain critical findings (99.9% expert-verified sensitivity). Second, a panel of blinded expert clinicians (R.J., A.P. and T.H.) then reviewed the flagged ground truth and generated reports to assign a ‘final, gold standard, triage decision’. Our prospective dataset included a total of 1,155 patients, with 155 patients having at least one critical finding (Supplementary Table 7).

**Fig. 5:**
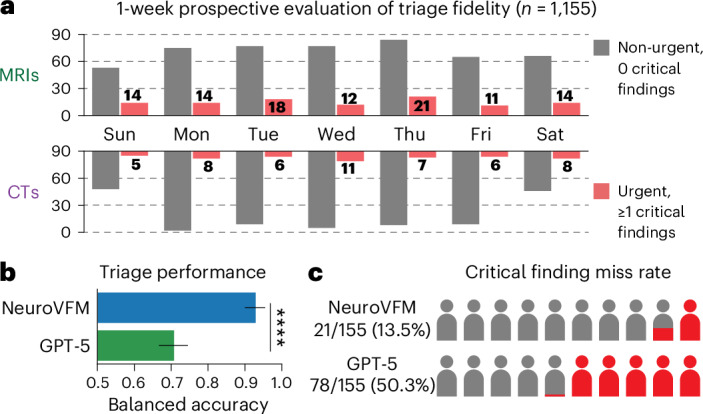
Prospective triage evaluation. **a**, Descriptive statistics for non-urgent versus urgent MRI and CT studies for the 1-week prospective window across the Michigan Medicine health system. **b**, NeuroVFM achieved a mean balanced accuracy of 92.6% (95% CI 89.8−95.2%) compared to 71.2% (95% CI 67.2−75.2%) for GPT-5, generated using bootstrapping (10,000 replicates); ****P < 0.0001, one-sided paired bootstrap. **c**, The critical finding miss rate for NeuroVFM was almost 4× lower than GPT-5. Notably, NeuroVFM leaves room for improvement as an autonomous screening tool due to a sensitivity of 86.5%, missing 21 of 155 patients with a critical finding.

The NeuroVFM arm achieved better critical findings and triage accuracy (92.6% balanced accuracy; 95% CI 89.8−95.2%), outperforming GPT-5 by +21.4% (71.2% balanced accuracy; 95% CI 67.2−75.2%; one-sided paired bootstrap, *P* < 0.0001; Fig. 5b and Supplementary Table 8). Errors in the NeuroVFM arm were primarily attributable to missed radiographic findings rather than clinician mis-triage (Supplementary Fig. 4). Among the 187 flagged NeuroVFM-generated reports, all urgent cases were labeled as urgent (134/134), with moderate over-triage among non-urgent cases (13/53). In all urgent misses (21/155), the NeuroVFM-generated report did not identify the critical finding, leading to the screening model not flagging the study, resulting in an overall sensitivity of 86.5% (95% CI 81.0−91.6%; Fig. 5c). These results demonstrate an important opportunity for future improvement of NeuroVFM-driven neuroradiology workflows.

## Discussion

Health systems are knowledge bases and data engines that capture the collective experience of public health and clinical medicine. Here we demonstrate that directly experiencing the clinical world through health system learning achieves high-performance neuroimaging foundation models. These task-agnostic vision models, pretrained on large and diverse clinical datasets, provide robust and transferable representations for downstream tasks. By integrating domain-specific perception learned from private clinical data with general-purpose reasoning, health system learning and NeuroVFM provide a roadmap toward AI systems that interpret and act on clinical information with expert reliability.

Frontier models benefit from the vastness of publicly available information; however, this breadth limits their depth in technical domains, such as medicine^18^. Internet data rarely reflect the complexity and diversity of real-world clinical imaging, where disease manifestations, acquisition protocols and patient anatomy can vary widely. Recent evidence demonstrates the potential risks of relying on proprietary, internet-trained LLMs for triage recommendations^19^. Health system learning represents a fundamentally different paradigm^20,21^. Rather than learning from descriptions of the clinical world or curated hand-selected patient examples, health system learners, such as NeuroVFM, experience the world itself, modeling the raw signatures of diseases embedded in unfiltered clinical data. World models have gained attention as a strategy to address the limitations of sequence modeling and reinforcement learning^22–24^.

Our long-term vision for NeuroVFM is to complement LLMs and frontier models, rather than replace them, in medicine. Agentic AI systems, including GPT-5 and Claude Sonnet 4.5, have integrated tool use to improve performance in specific domains, such as mathematics, science and medicine^25–27^. We foresee NeuroVFM and other health system models being integrated into agentic AI systems as external modules that provide a grounded interpretation of clinical data. Frontier models will be able to reason over privacy-preserving, structured, clinically calibrated outputs from NeuroVFM, reducing failure modes and providing an auditable substrate for action. We think that expert-level performance across most cognitively complex domains will require the integration of domain-specific modules into general-purpose AI models.

Limitations and directions for future research include the need to integrate temporal and additional multimodal data streams, including pathology, genomics and longitudinal clinical outcomes, to construct unified representations of disease progression. The present study focuses on neuroimaging, but the unified Vol-JEPA architecture is inherently extensible to other medical imaging modalities and body parts. Finally, although NeuroVFM exhibits interpretability, translating these insights into actionable clinical interfaces will require new methods for human−AI collaboration, uncertainty quantification and prospective clinical studies across multiple health systems.

In summary, NeuroVFM demonstrates that generalist AI models can be built from the health system itself. Learning directly from health system data yields representations that translate to diagnosis, triage and report generation. We provide a scalable blueprint toward the development of medical foundation models that will be transformative in 21st century healthcare.

## Methods

### Health system learning

We define ‘health system learning’ as learning directly from uncurated data generated during routine clinical operations. This is distinguished from internet-scale pretraining, which lacks access to private clinical data, and from report-supervised health system pretraining, which is bottlenecked by requiring paired reports and what they capture. Health system learning can also be contrasted with standard machine learning pipelines that include data selection for a specific disease—for example, brain tumor MRIs or patients with Alzheimerʼs disease—and subsequently training a classifier to diagnose that disease. Health system learners are trained without a specific diagnosis or classification as a target. We also clarify that health system learning does not imply learning from ‘all data’ within a health system. Rather, health system learning is the environment within which generalist medical foundation models can emerge^29^. We introduce NeuroVFM as the imaging-first instantiation of this approach on clinical neuroimaging CT and MRI scans.

### UM-NeuroImages dataset

Radiology studies at large academic medical centers are stored in picture archiving and communication systems (PACSs). We queried the University of Michigan PACS via an SQL-based interface (Sectra Data Warehouse (SDW)) for neuroimaging studies satisfying three criteria: (1) acquisition data prior to 1 June 2024; (2) an examination of body part(s) including the head, brain, orbits, face or neck; and (3) a modality of MRI or CT. The specific query parameters are in Supplementary Fig. 5.

The initial query yielded 645,989 unique studies. We excluded 79,074 entries that contained non-image objects (for example, PDFs and screenshots) or corrupted Digital Imaging and Communications in Medicine (DICOM) files. The final cohort comprised 566,915 studies (275,981 MRI; 290,934 CT) totaling 5,239,579 series (3,647,950 MRI; 1,591,629 CT). We split the data temporally: studies acquired before 1 June 2023 formed the training set, and studies from 1 June 2023 through 31 May 2024 formed the test cohort. We only included studies with reports for the test set, resulting in 21,054 CT studies and 29,239 MRI studies. The full training set was used for self-supervised pretraining of the NeuroVFM encoder, which requires no labels or reports. All downstream supervised evaluations used the 444,188 training studies with paired radiology reports (219,882 MRI; 224,306 CT). From this report-paired subset, we held out 991 MRI studies and 722 CT studies as a validation set for hyperparameter tuning.

Labels were derived from radiology reports using an LLM-based extraction pipeline, and a stratified subset was manually reviewed by clinical experts. We first define a clinically organized ontology of 82 CT and 74 MRI diagnoses (Extended Data Fig. 2h). We then used GPT-4.1-mini to extract these 156 diagnoses from radiology reports and to generate structured summaries of key findings, following previous report-parsing work^13,30–32^. Prompts used are in Supplementary Figs. 6−8. To assess annotation quality, two neuroimaging experts (R.J. and T.H.) independently reviewed a stratified sample drawn across all diagnostic labels. For each label, we selected two pipeline-positive and two pipeline-negative studies, providing the label, report text and clinical indication. Reviewers were blinded to pipeline predictions and determined whether the label was supported by the accompanying report and indication. Annotation performance metrics are reported in Supplementary Table 1.

### Series preprocessing

To ingest the full breadth of health system data without manual curation, we applied an automated pipeline to standardize intensities and dimensions. Series were resampled to 1 × 1 × 4 mm (4 mm along the acquisition axis from DICOM) and saved as an 8-bit uint. MRI intensities were clipped to the 0.5−99.5th percentile before quantization. CT volumes yielded three windows: brain (width = 80, level = 40; 8-bit), subdural/blood (width = 200, level = 80; 4-bit) and bone (width = 2,800, level = 600; 4-bit). The 4-bit windows reduced storage while preserving contrast for key pathologies. Background masks were derived via Otsu and Hounsfield thresholding for MRI and CT, respectively. During training, volumes were cast to float32, scaled to [0,1] and mean normalized using statistics derived from the training set. Extended Data Fig. 1b summarizes this pipeline.

### NeuroVFM training with Vol-JEPA

The core training objective for NeuroVFM was large-scale self-supervision, applied at the individual volume level, across the entire UM-NeuroImages dataset. NeuroVFM employs a JEPA to optimize a masked modeling objective directly in the representation space. First, input volumes are tokenized into non-overlapping 3D patches of 4 × 16 × 16 voxels. Each 3D volume is then partitioned into a small, visible context region, *x*, and a larger, masked target region, *y*. The tokenized context region is processed by a trainable student encoder, *E*_*θ*_. The resulting context representations, *E*_*θ*_(*x*), along with positional information of the masked target patches, are then fed to a predictor module, *P*_*ϕ*_, which generates predictions for the representations of these target patches. The complete set of tokens from the input volume (representing both context and target regions) is processed by a teacher encoder, whose weights, ${E}_{\bar{\theta }}$, are updated as an exponential moving average (EMA) of the student encoderʼs weights after each step and are not directly optimized by gradient descent. The training objective minimizes the difference between the predicted representations of the target patches and those generated by the teacher network for the same target patches. This is formulated as^5^:

$${\mathrm{minimize}}_{\theta ,\phi ,{\Delta }_{y}}\parallel {P}_{\phi }({\Delta }_{y},{E}_{\theta }(x))-{\mathrm{sg}}({E}_{\bar{\theta }}(y)){\parallel }_{{\mathrm{smooth}}{\rm{L}}1},\,$$

where Δ_*y*_ is a set of learnable tokens that represents the masked target patches, and sg( ⋅ ) denotes a stop-gradient operation. This representation-level objective is computationally efficient and encourages the learning of semantic features without requiring explicit voxel-level augmentations or decoding.

Vol-JEPA extends the principles of I-JEPA^5^ and V-JEPA^7^ for self-supervision to volumetric neuroimages, predicting representations of masked 3D target patches based on visible 3D context patches. This encourages the model to learn the shared anatomy of the brain, head and neck. The masking strategy was foreground focused: context and target patches were sampled exclusively from the precomputed head mask. Masking was performed in two ways: (1) multiple large crops are sampled, with their union forming the masked target, and (2) a small crop is sampled as context, with the complement being the target. A random subset of context patches is then dropped, serving as additional target patches to predict. Unlike approaches that truncate sequences within a mini-batch to a uniform length, our implementation leverages FlashAttention-2 (ref. ^33^) to directly encode variable-length sequences of context and target patches derived from input volumes. To ensure robustness to varying patient orientations, we applied random axis permutations and flips to each volume, using the same transform for both student and teacher inputs. A hyperparameter search indicated optimal context region sampling ratios (from the total patches within a given crop) are 25% for MRI volumes and 20% for CT volumes, with a patch dropout rate of 20%. An overview of this masking strategy can be found in Extended Data Fig. 3a.

During training, volumes were truncated along each axis to a maximum of 20 patches to bound the token count per crop. At inference, the same encoder was applied to the entire volume without truncation, leveraging its ability to handle variable-length token sequences. For CT scans, which are typically viewed with multiple windowing presets (for example, brain, subdural and bone), we implemented a weighted sampling strategy during training. For pretraining, one of these three CT window settings was randomly selected and applied with probabilities of 0.7 (brain), 0.15 (subdural) and 0.15 (bone), and all windows were used during inference. Full training details are in Supplementary Table 9.

### Grounded diagnoses with multiple instance learning

Most visual grounding evaluation requires an object detection module to output a bounding box around an image region given a label or text prompt. This is not feasible for the present study because there are no object detection datasets or models sufficiently powerful to evaluate neuroimage grounding on the scale and complexity of UM-NeuroImages. Because most neurologic pathologies are spatially small relative to the full study, study-level labels provide only weak supervision. We leveraged an attention-based multiple instance learning (AB-MIL) framework to assess neuroimage grounding^34^. AB-MIL is known to assign high attention to diagnostic regions in medical images^35^. Unfortunately, the standard AB-MIL framework, with its ‘aggregate-then-classify’ design, cannot resolve a critical grounding ambiguity: it is unable to disentangle a patch’s importance (‘where’ the model looks) from its directional contribution (‘why’ it is considered positive or negative evidence).

We address this gap with a pooling operation that reverses the standard order to a ‘classify-then-aggregate’ model. For a task with *K* classes and a bag of *N* instances, we first compute classification logits for each instance *i* using a multilayer perceptron (MLP), *ψ*_*p*_. A separate attention MLP, *ψ*_*m*_, generates *K* class-specific attention scores per instance. For each class, these scores are normalized with a softmax across instances to obtain class-specific attention weights. The final bag-level logits, *p*(*x*), are the sum of the elementwise product of the per-instance logits and their corresponding attention weights:

$$p(x)=\mathop{\sum }\limits_{i=1}^{N}{\alpha }_{i}\circ {\psi }_{p}(f({x}_{i}))$$

Here, *f*(*x*_*i*_) is the frozen feature vector for instance *i*; *α*_*i*_ is the vector of class-specific attention weights; and ∘ denotes the Hadamard product. This formulation yields interpretable, label-specific attention maps that reflect both the importance and directional contribution of each region to the diagnostic decision, providing a scalable means of evaluating grounding without region-level annotations.

### Comparison to foundation model baselines

We evaluated NeuroVFM against two families of baselines: (1) internet-scale pretrained encoders and (2) methods directly trained on UM-NeuroImages. All backbones were frozen and evaluated with the same study-level attentive probe and data splits. Full architecture details, training hyperparameters and evaluation configurations can be found in Supplementary Table 9 and in our GitHub repository. A schematic of the study-level pooling strategies can be found in Extended Data Fig. 1e.

#### Internet-scale pretrained encoders

We chose two baselines representative of the dominant internet-scale paradigms:

- Natural image self-supervision, DINOv3 (ref. ^9^): ViT-B/16 pretrained on 1.7 billion natural images (HuggingFace, https://huggingface.co/facebook/dinov3-vitb16-pretrain-lvd1689m).
- Medical image−text alignment, BiomedCLIP^10^: ViT-B/16 pretrained on 15 million image−caption pairs scraped from PubMed (HuggingFace, https://huggingface.co/microsoft/BiomedCLIP-PubMedBERT_256-vit_base_patch16_224).

For CT studies, all baselines were provided the same set of windowed series as NeuroVFM (with brain, blood and bone windows given as separate series). 3D volumes were processed slicewise according to the modelʼs respective preprocessing pipeline (for example, DINOv3 necessitates resizing to 224 × 224 and normalizing with ImageNet mean/s.d.), with slice-level features aggregated to produce study-level predictions.

#### Methods trained on UM-NeuroImages

To isolate the effect of pretraining objective from data, we trained several baseline architectures on UM-NeuroImages spanning the following paradigms: voxel reconstruction (MAE-style), self-distillation (DINO-style) and study report alignment (CLIP-style). For voxel reconstruction, we trained NeuroMAE, which uses the same 3D ViT encoder, pretraining data and optimization schedule as NeuroVFM but replaces the latent prediction objective with voxel reconstruction^36^. We adjusted the decoder size and masking ratio, using 85% foreground-masking random masking. This pairing directly isolates the effect of the pretraining objective. For self-distillation, we trained a DINOv2 model^11^ on two-dimensional (2D) slices derived from UM-NeuroImages volumes. For report-supervised pretraining, we trained HLIP^8^, a 3D medical vision-language model, on 444,188 study−report pairs from UM-NeuroImages and evaluated its frozen image encoder. HLIP training mini-batches were balanced across MRI and CT studies, and the CLIP objective was computed within each modality to prevent trivial cross-modality discrimination. We additionally benchmarked PRIMA^4^, a 3D vision-language model trained on the MRI subset of UM-NeuroImages, as a second report-supervised baseline.

### Diagnostic evaluation of NeuroVFM

The UM-NeuroImages dataset contains two decades of clinical neuroimaging data from a large academic health system, spanning a broad spectrum of neurological presentations. To evaluate ‘imaging-only’ diagnostic ability, we tested NeuroVFM on a temporally held-out test cohort beginning immediately after the pretraining window. All encoders were frozen, and modality-specific attentive probes were trained for multi-label prediction using class-weighted binary cross-entropy. We prespecified a primary endpoint of macro-averaged AUROC across all 156 diagnostic tasks and a secondary endpoint of per-category AUROC. For the primary endpoint, pairwise differences between models were assessed by paired, study-level bootstrap (10,000 replicates). For the secondary endpoint, category-level comparisons were corrected for multiple hypotheses using Benjamini−Hochberg correction at *q* < 0.05. Hyperparameters and class-specific thresholds were selected on the validation set. We report per-class AUROC, balanced accuracy, sensitivity and specificity on the test cohort, with 95% CIs from study-level bootstrap resampling. Full results are provided in Supplementary Tables 10 and 11.

Because the split is temporal rather than patient level, patients who received imaging in both periods appear in both training and test sets (34.7% of MRI and 31.8% of CT test set patients). This mirrors the intended deployment setting, where a model trained on historical data encounters both new and returning patients. To verify that this overlap does not inflate performance, we repeated all evaluations after removing studies from overlapping patients. Aggregate performance and model rankings were largely unchanged (Supplementary Table 12).

#### Data, model and modality scaling

We studied performance along data scale, model capacity and modality with resource-normalized protocols. For modality, we held the optimization budget fixed at approximately 510,000 training steps and compared (1) a single multimodal model versus (2) two unimodal models (one MRI, one CT) trained separately. For data and model scaling, we varied (1) the fraction of UM-NeuroImages (5%, 10%, 25%) and (2) the encoder size (ViT-Small, ViT-Medium, ViT-Base). Larger backbones (for example, ViT-Large) were left to future work due to computationally prohibitive hyperparameter search.

#### Label efficiency

We quantified how per-task performance scales with the amount of supervision. For each diagnostic class *c* and modality *m* ∈ {CT, MRI}, we calibrated model probabilities on the validation set using Platt scaling and computed F1 on the held-out test set. We excluded classes with fewer than 30 training positives (*n*_pos_) and 10 testing positives. We fit a simple linear model of F1 versus ${\log }_{10}({n}_{{\rm{pos}}})$ via ordinary least squares (OLS) with heteroskedasticity-consistent standard errors of type 3 (HC3 SEs). Modality fixed effects and interactions assessed CT versus MRI differences. Encoder comparisons (NeuroVFM versus HLIP, DINOv3 and BiomedCLIP) used ANCOVA to test slope equality. If interaction terms were non-significant, a shared slope was used; otherwise, encoder-specific slopes were retained. Label equivalence was reported as the fold increase in positives that a baseline requires to match NeuroVFM at a fixed F1, with 95% CIs from 10,000 bootstrap replicates over classes.

### NeuroVFM on external public benchmarks

To assess out-of-distribution generalization, we evaluated frozen NeuroVFM performance on eight public neuroimaging benchmarks. For all tasks, we trained an attentive probe without updating the encoder. Within each dataset, we held out 20% of patients as a stratified test set. On the remaining 80%, we performed eight-fold iterative stratified cross-validation to select probe hyperparameters. The eight probes trained on the cross-validation folds were then used to generate logits on the held-out test set, which were averaged per study to form the final ensemble. All reported metrics include 95% study-level boostrapped CIs (10,000 replicates). This approach rigorously tests the quality of the learned representations and, through the probeʼs attention weights, allows for the identification of class-discriminative tokens for each task.

#### External MRI benchmarks

We evaluated NeuroVFM on six public MRI datasets, spanning a range of neurological and psychiatric conditions. On the multi-site OpenBHB dataset^37^, we benchmarked brain age regression to test for fine-grained anatomical representation. For dementia-related tasks, we used the ADNI dataset^38^ to perform cognitively normal versus Alzheimerʼs disease classification and to distinguish progressive from stable mild cognitive impairment (sMCI versus pMCI), with a 20% stratified test set held out within ADNI. We then evaluated the ADNI-trained Alzheimerʼs disease classifier externally on the OASIS-1 (ref. ^39^) and AIBL^40^ datasets, applying the frozen probe without further fine-tuning to distinguish cognitively normal individuals (Clinical Dementia Rating (CDR) = 0) from those with dementia (CDR ≥ 1). We further evaluated diagnostic classification on several consortium datasets: differentiating individuals with autism spectrum disorder from typically developing controls on the ABIDE dataset^41^ and Parkinsonʼs disease from healthy controls on the PPMI dataset^42^.

#### External CT benchmarks

Performance on detecting critical neuroradiological findings was evaluated on two public non-contrast head CT cohorts, selected to test generalization on both a large-scale challenge dataset and a smaller, deeply annotated clinical cohort. The large-scale 2019 RSNA-ICH challenge dataset^43^ was used to benchmark multi-label classification of intracranial hemorrhage and its five subtypes (epidural, intraparenchymal, intraventricular, subarachnoid and subdural). The high-quality, expert-annotated CQ500 dataset^44^ was used for a more extensive evaluation across 14 diagnostic labels, including detailed hemorrhage characterization (subtypes, laterality, chronicity), skull fracture detection and signs of structural abnormality (for example, mass effect and midline shift).

### Vision instruction tuning for radiology report generation

To evaluate the potential of NeuroVFM as a visual backbone for multimodal applications, we adapted a LLaVA-1.5-style visual instruction tuning framework^16^. This experiment was designed not to optimize report generation performance but, rather, to assess the feasibility of coupling NeuroVFMʼs learned representations with an LLM with minimal architectural modifications.

#### NeuroVFM-LLaVA architecture

NeuroVFM-LLaVA comprises three components: (1) the frozen NeuroVFM visual encoder, (2) an open-source LLM (Qwen3-14B)^15^ and (3) a connector module to bridge them.

Standard LLaVA connectors use a two-layer MLP to project visual features into the LLMʼs word embedding space. This approach is insufficient for the high dimensionality of multi-sequence neuroimaging, where a single study may comprise a large and variable number of visual tokens (≥20,000). To address this, our connector module first employs a Perceiver-style architecture that operates sequencewise (for example, on T1, T2 and FLAIR independently)^45,46^. The Perceiver aggregates the variable-length token sequence from each scan into a fixed-length representation of 64 latents. These fixed-length latents (total latents = 64× num_sequences) are then concatenated and passed to a two-layer MLP projector.

#### Training dataset and curation

The training dataset was derived from 444,188 unique neuroimaging studies. To create the text pairs, original radiology reports were summarized using GPT-4.1-mini to extract a concise list of key radiological findings.

Acknowledging that naive training on imbalanced medical datasets can degrade performance, we curated the training set via data resampling. Using the previously extracted diagnostic labels from each report, we performed weighted random sampling with replacement, assigning each study a weight inversely proportional to the prevalence of its rarest associated label. This process, which aims to balance the representation of less common pathologies, resulted in the final training dataset of approximately 270,000 unique image−text pairs (444,188 total including duplicates).

#### Training strategy

Following the LLaVA methodology, training proceeded in two stages.

1. Stage 1 (Connector Pretraining). Only the connector module (Perceiver and MLP) weights are updated. The model was trained to map NeuroVFM image features to the corresponding reference key findings, which were formatted as a single, concatenated string.
2. Stage 2 (Full Fine-tuning). Both the connector and the LLM weights are updated. In this stage, the model was trained on an instruction-following task. The input prompt was fixed to: ‘Generate a concise report of the key positive findings for this study.’ The target output was the same set of findings but formatted as a structured JSON list ordered by clinical relevance.

Further details on training hyperparameters are provided in Supplementary Table 13.

### Evaluation of preliminary report generation

We evaluated NeuroVFM-LLaVA against two proprietary multimodal frontier models: GPT-5 (gpt-5-2025-08-07, ‘reasoning’ medium, ‘verbosity’ low) and Claude Sonnet 4.5 (claude-sonnet-4-5-20250929, ‘thinking’ enabled). We additionally evaluated MedGemma 1.5 4B, an open-source healthcare MLLM by Google DeepMind. This evaluation assessed two criteria: (1) the factual accuracy of generated findings and (2) the clinical utility of these findings for a downstream triage task. To support this evaluation, we established business associate agreements (BAAs) with vendors offering commercially available Health Insurance Portability and Accountability Act (HIPAA)-compliant access to frontier models, specifically OpenAI and Anthropic. This enabled the secure exchange of protected health information (PHI) while preserving patient privacy. The prompt used to generate study findings using frontier models can be found in Supplementary Fig. 9.

#### Benchmark model adaptation (3D-to-2D conversion)

A primary challenge in benchmarking against proprietary models is that they accept only 2D images and have limitations on total input image tokens. To create a fair comparison with our 3D-native model, we developed a systematic 3D-to-2D conversion pipeline.

1. All 3D volumes (preprocessed with the NeuroVFM pipeline) were converted into 224 × 224-pixel 2D slices by resampling along the axis of acquisition and keeping every other slice. For MedGemma 1.5, CT studies were processed as three-channel 2D slices with each channel representing a different windowing, as per their specifications^47^.
2. Non-diagnostic and derived sequences (for example, scout images and phase/magnitude maps) were excluded.
3. Due to application programming interface (API) token limits, inputs were constrained to 15 sequences per study for GPT-5 (approximately 360 slices) and four for Claude Sonnet 4.5 (approximately 96 slices). For MedGemma 1.5, we provided two sequences per study as we found that led to optimal performance.
4. If a study exceeded this limit, sequences were deterministically prioritized based on clinical relevance (that is, for MRI: post-contrast T1, diffusion-weighted imaging/apparent diffusion coefficient (DWI/ADC), FLAIR, susceptibility-weighted imaging (SWI), T2, non-contrast T1; for CT: at least one brain, blood and bone window).
5. All selected slices were converted to float and normalized between [0,1], and slices with more than 90% black pixels were dropped.

Increasing from five to 15 series per study improved three-tier acuity accuracy only marginally on both MRI and CT, indicating that performance differences between models were not driven by input volume (Extended Data Fig. 9e).

#### Expert evaluation of generated reports

All generative models were evaluated on ‘UM-NeuroImages-Acuity’, a new, manually curated test set of 600 studies (300 validation, 300 hold-out) derived from our test set. This set was hand-selected by neuroimaging experts to be balanced across three acuity classes (‘Unremarkable’, ‘Routine’, ‘Urgent’) and two modalities (MRI, CT). This allows for estimation of class-conditional safety metrics rather than prevalence-weighted deployment performance.

We assessed report quality through automated metrics, BLEU-2 (ref. ^48^), ROUGE-L^49^ and METEOR^50^, as well as with human evaluation. To ensure effective blinding, all model outputs were standardized to a uniform text format. For NeuroVFM-LLaVA and GPT-5, a neuroimaging expert (T.H.) performed a structured review of each study to quantify (1) capture of key findings, (2) clinically significant hallucinations and (3) laterality errors. To evaluate preferences, three neuroimaging experts from the United States and Europe (R.J., A.-K.M. and T.H.) then performed a blinded, randomized pairwise review comparing generated findings from NeuroVFM-LLaVA and GPT-5 against the ground truth clinical report. For each case, model outputs were randomly labeled ‘Report A’ and ‘Report B’. Evaluators selected their preferred report (‘Report A’, ‘Report B’ or ‘Both’) based on overall clinical utility for acuity assessment. Because Claude Sonnet 4.5 and MedGemma 1.5 both exhibited substantially lower acuity accuracy on UM-NeuroImages-Triage, we restricted expert review and preference testing to NeuroVFM-LLaVA and the strongest baseline (GPT-5).

To quantify the clinical utility of the generated findings, we employed an ‘LLM-as-a-judge’ pipeline. In each analysis, we designated a frontier model (GPT-5 or Claude Sonnet 4.5) as a separate ‘acuity assessor’, instructed with a strict, predefined set of criteria (Supplementary Fig. 1). We first confirmed that each LLM achieved high accuracy when classifying acuity from the ground truth radiology report alone. The LLM was then prompted to classify each study as ‘Unremarkable’, ‘Routine’ or ‘Urgent’ based solely on the generated text findings from each model (NeuroVFM-LLaVA, GPT-5 and Claude Sonnet 4.5) and the clinical indication. Using GPT-5 versus Claude Sonnet 4.5 as the acuity assessor yielded similar results, indicating that the protocol is model agnostic and not biased toward any single frontier model. This procedure converts report generation into a three-class classification problem and provides a quantitative measure of clinical utility.

### Prospective feasibility study of report sufficiency for triage

We conducted a prospective silent evaluation over a consecutive 1-week window (18−25 January 2026), including all CT and MRI studies of the head, brain, face, neck and orbits performed during routine care (*n* = 1,155; 601 MRIs and 544 CTs). No additional curation or exclusion criteria were applied beyond modality and anatomic region. For each study, we generated a structured report using (1) NeuroVFM-LLaVA or (2) GPT-5, a multimodal baseline demonstrated to be the best model at findings generation in Fig. 4. Reviewers were blinded to the source of the report (ground truth, NeuroVFM, GPT-5) when evaluating.

To approximate a workload-aware triage workflow, we used a fixed two-stage process. First, a frontier reasoning model (GPT-5-thinking with a prespecified prompt and temperature) screened each report and assigned a binary decision: ‘flag for clinician review’ or ‘do not flag’. Second, for all flagged studies, a panel of imaging-centric clinicians assigned a final, gold standard triage label (urgent versus non-urgent) based solely on the model-generated report and clinical indication. Specifically, two clinicians (T.H. and A.P.) independently annotated flagged studies, and the third expert (R.J.) adjudicated any discordant labels. Urgent cases were defined as studies containing findings that would reasonably warrant escalation or intervention. To assess whether the screening step was failing to capture urgent studies, we additionally audited all unflagged studies, with all but one being non-urgent (99.9% sensitivity). This study was moved into the flagged subset for final evaluation. Each study was assigned a reference urgency label derived from the ground truth radiology report.

The primary analysis evaluated whether generated reports were sufficient to support accurate triage under the two-stage workflow. We quantified (1) the fraction of studies flagged for review (workload proxy), (2) urgent miss rate attributable to the screening stage (urgent studies not flagged), (3) triage performance among flagged studies and (4) overall triage performance (balanced accuracy and urgent miss rate). For each metric, we computed 95% CIs using study-level bootstrapping (10,000 replicates).

### Computational hardware and software

All data used to train NeuroVFM were acquired over more than two decades of routine clinical care at Michigan Medicine. Studies were identified in the Michigan Medicine PACS via the SDW. The aggregate size of raw Neuroimaging Informatics Technology Initiative (NIfTI) volumes is approximately 150 TB, stored on the Advanced Research Computing (ARC) DataDen object storage service. Postprocessing (removal of non-image/corrupt entries and embedded metadata, background removal and quantization) reduced the footprint by over an order of magnitude to approximately 9 TB. Preprocessed volumes were written as large NumPy memory-mapped shards (10 shards for the training set; one each for the validation and test sets) with JSON index manifests to enable zero-copy random access during training.

All experiments were executed on the University of Michigan ARC Armis2 cluster using Simple Linux Utility for Resource Management (SLURM). Typical jobs used nodes with eight NVIDIA L40S GPUs (48 GB each), 64 Intel Xeon Platinum 8358 CPU cores and 503 GB RAM. Vol-JEPA pretraining used PyTorch Distributed Data Parallel (DDP), whereas LLM fine-tuning used PyTorch Fully Sharded Data Parallel (FSDP). A full Vol-JEPA pretraining run required fewer than 1,000 GPU hours in aggregate (batch size of 768, approximtely 510,000 steps), making the approach feasible on university-hosted compute clusters. Training used automatic mixed precision (AMP) with fixed random seeds.

For single-volume inference in bfloat16, peak GPU memory ranges from 0.89 GB (ViT-Small, 21.7 million parameters) to 1.10 GB (ViT-Base, 85.8 million parameters) on a single L40S, with throughput of 84 volumes per second across all encoder sizes. Models were implemented in Python 3.10.14 with PyTorch 2.5.0 (CUDA 12.4). Vol-JEPA pretraining and diagnostic probing used PyTorch Lightning 2.5.0.post0. All data handling and computation occurred on HIPAA-compliant ARC infrastructure. Model weights are available under the CC-BY-NC-SA-4.0 license, enabling the broad dissemination of learned clinical representations without the risk of PHI leakage.

### Use of LLMs

LLMs were used to assist with code prototyping and manuscript editing. All analyses, text and code were reviewed and verified by the authors, who take full responsibility for the work.

### Ethical, regulatory, governance and inclusion statement

Our research was approved by the University of Michigan institutional review board (IRB) (HUM00229133). All MRI and CT data were acquired under secondary data usage. The methods were carried out in accordance with the IRBʼs guidelines, regulations and policies. All human subjects who met inclusion criteria as stated above were included in the study. No treatment decisions were informed by NeuroVFM, as our model is not approved by the US Food and Drug Administration. Our model is susceptible to bias secondary to training dataset, model architecture and objective functions. NeuroVFM should be used as a research tool only.

### Reporting summary

Further information on research design is available in the Nature Portfolio Reporting Summary linked to this article.

## Online content

Any methods, additional references, Nature Portfolio reporting summaries, source data, extended data, supplementary information, acknowledgements, peer review information; details of author contributions and competing interests; and statements of data and code availability are available at 10.1038/s41591-026-04497-1.

## Supplementary information

Supplementary Information Supplementary Figs. 1−9 and legends for Tables 1−13.

Reporting Summary

Peer Review File

Supplementary Table 1 Included in ‘NeuroVFM_Supplementary_Information.pdf’.

Supplementary Table 2 Included in ‘NeuroVFM_Supplementary_Information.pdf’.

Supplementary Table 3 Included in ‘NeuroVFM_Supplementary_Information.pdf’.

Supplementary Table 4 Included in ‘NeuroVFM_Supplementary_Information.pdf’.

Supplementary Table 5 Included in ‘NeuroVFM_Supplementary_Information.pdf’.

Supplementary Table 6 Included in ‘NeuroVFM_Supplementary_Information.pdf’.

Supplementary Table 7 Included in ‘NeuroVFM_Supplementary_Information.pdf’.

Supplementary Table 8 Included in ‘NeuroVFM_Supplementary_Information.pdf’.

Supplementary Table 9 Included in ‘NeuroVFM_Supplementary_Information.pdf’.

Supplementary Table 10 Included in ‘NeuroVFM_Supplementary_Information.pdf’.

Supplementary Table 11 Included in ‘NeuroVFM_Supplementary_Information.pdf’.

Supplementary Table 12 Included in ‘NeuroVFM_Supplementary_Information.pdf’.

Supplementary Table 13 Included in ‘NeuroVFM_Supplementary_Information.pdf’.

## Data Availability

IRB approval was obtained from the University of Michigan for MRI data collection. Restrictions apply to the availability of raw patient MRI and CT imaging data, which were used with institutional permission through IRB approval for the present study and, thus, are not publicly available. All data sharing among medical centers is regulated through data use agreements with the study authors. A similar data-sharing protocol may be established for interested investigators. Please contact the corresponding author (T.H.) for any requests for data sharing. All requests will be evaluated based on institutional and departmental policies to determine whether the data requested are subject to intellectual property or patient privacy obligations. Data can be shared only for non-commercial academic and investigational purposes.

## Code availability

All code was implemented in Python (version 3.10.14) using PyTorch (2.5.0) compiled with CUDA 12.4 as the primary machine learning framework. The following packages were used for data preprocessing, model training and evaluation: pydicom (2.4.4), nibabel (5.3.2), SimpleITK (2.4.0), torchvision (0.20.0), pandas (2.2.3), NumPy (2.1.2), PyTorch Lightning (2.5.0.post0), flash-attn (2.6.3), matplotlib (3.10.7), scipy (1.15.2) and scikit-learn (1.6.1). The following packages were used to load baselines: open-clip (2.23.0) and transformers (4.56.0). All code and scripts to reproduce the experiments in this study are available on GitHub at https://github.com/MLNeurosurg/neurovfm under an MIT license.
