# Supplementary material for: Health system learning enables generalist neuroimaging models
Source: Nat Med. 2026 Jul 10;32(8):2831–7. doi: 10.1038/s41591-026-04497-1 (PMC13472962; doi:10.1038/s41591-026-04497-1)
Supplement: Supplementary file 1 [file 41591_2026_4497_MOESM1_ESM.pdf]

nature medicine

Supplementary information

https://doi.org/10.1038/s41591-026-04497-1

Health system learning enables generalist neuroimaging models

In the format provided by the authors and unedited

Prompt: Assign triage level given findings and indication

```
# Objective:
Analyze the provided list of radiological findings from a neuroimaging study, using the provided clinical indication to inform your
analysis, and generate a clinical triage analysis as a single JSON object.

# Instructions:
1.  Assess: Write a single, concise "triage_assessment" sentence which explains the clinical acuity of the patient's condition based
on the provided findings. Your assessment should be based ONLY on this text. Do not infer any information not present in the list.
2.  Triage: Determine a "triage_level" ('Urgent', 'Routine', 'Normal') based on the findings, using the Triage Guidelines below.

# Triage Guidelines:
- **Urgent**: A study is classified as 'Urgent' if it contains any new or worsening findings that require immediate clinical attention to
prevent significant harm.
- **Routine**: A study is classified as 'Routine' if it contains any notable findings that may require medical follow-up but are NOT
immediately life-threatening. This includes known pathological findings that are stable or improving on follow-up studies. Only
select Routine if the case REQUIRES specific clinical followup.
- **Normal**: A study is classified as 'Normal' if it is grossly unremarkable and does NOT require ANY clinical follow-up. Mild
mucosal thickening, mild chronic microvascular changes, mild calcifications, or mild cerebral volume loss are all considered Normal.

- **Examples of Urgent Findings:** (NON-exhaustive list meant to guide decision making)
  - Acute intracranial hemorrhage (e.g., subarachnoid, subdural, epidural, intraparenchymal)
  - Traumatic injury: Skull fractures (especially depressed or crossing vascular structures) or significant intracranial air (pneumocephalus)
  - Acute spinal column or cord injury: Vertebral fracture with subluxation, epidural hematoma, or severe cord compression
  - Signs of brain herniation or significant mass effect
  - Severe hydrocephalus or ventriculomegaly
  - Acute ischemic infarct (e.g., loss of grey-white differentiation, dense vessel sign) or large vessel occlusion on CT Angiography
  - Acute vascular injury, such as arterial dissection
  - Acute venous sinus thrombosis
  - Features of an aggressive mass (e.g., destructive bone lesion, ring-enhancing mass)
  - Features of severe infection (e.g., cerebral abscess, ventriculitis, encephalitis, epidural abscess)

# Output Format
- JSON object with 2 fields:
{
  "triage_assessment": "...",
  "triage_level": "Normal/Routine/Urgent",
}

Now, synthesize the given radiological findings and clinical indication into a triage assessment.
```

Supplementary Data Figure 1 | **Triage prompt given findings and indication.** Using a standardized protocol, we supply the clinical indication and itemized findings to a reasoning model (e.g., GPT-5-thinking) and request a single triage label with a brief justification. Labels are drawn from an expert-defined schema aligned with routine clinical triage. When provided ground-truth reports, both models show high accuracy and close agreement with clinician labels (Extended Data Fig. 9b), supporting the validity of their reasoning.

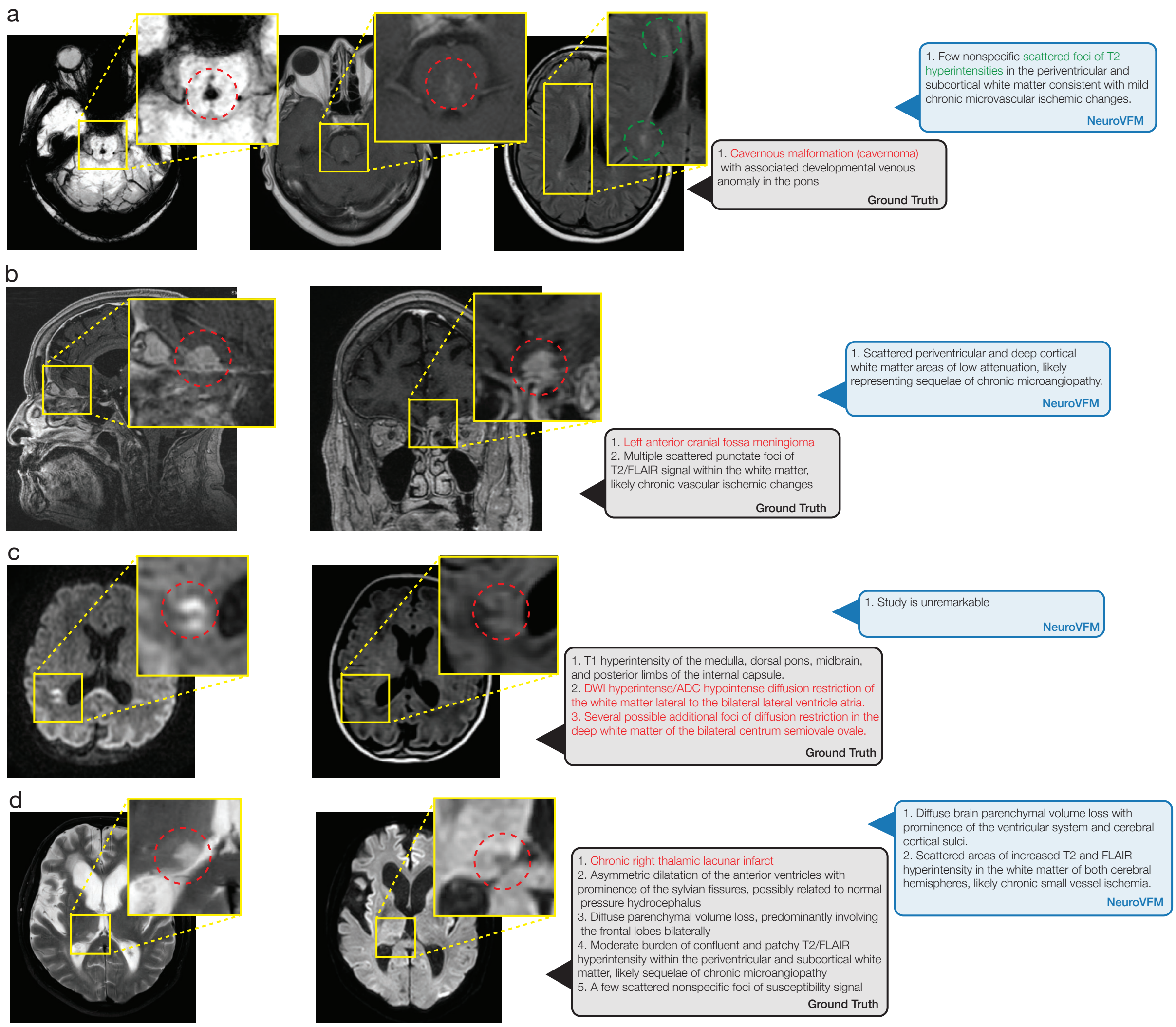

Supplementary Data Figure 2 | **NeuroVFM error analysis. a**, An example of a small brainstem cavernoma captured on susceptibility weighted imaging (SWI) (left). NeuroVFM missed this lesion due to the challenge of interpreting SWI images and the relatively small size of the cavernoma. Moreover, the lesion is poorly visualized on T1-weighted post-contrast images (middle). NeuroVFM was able to correctly identify small areas of microvascular changes on FLAIR. **b**, An example of a small olfactory groove meningioma. NeuroVFM fails to detect this lesion due to small size and location, with multiple other contrast-enhancing structures adjacent to the lesion, such as the cribriform and sinus mucosa. **c**, A neonate with evidence of hypoxic-ischemic brain injury. NeuroVFM struggles to identify small DWI hyperintense lesions both due to size and non-standard image processing of diffusion weighted imaging. **d**, A small chronic right thalamic lacunar infarct was correctly identified by the radiologist. NeuroVFM was able to identify several of the other chronic ischemic changes in the brain, but smaller, focal, ischemic lesions are often missed. We believe that errors associated with lesion size will be improved with smaller or adaptive token sizes.

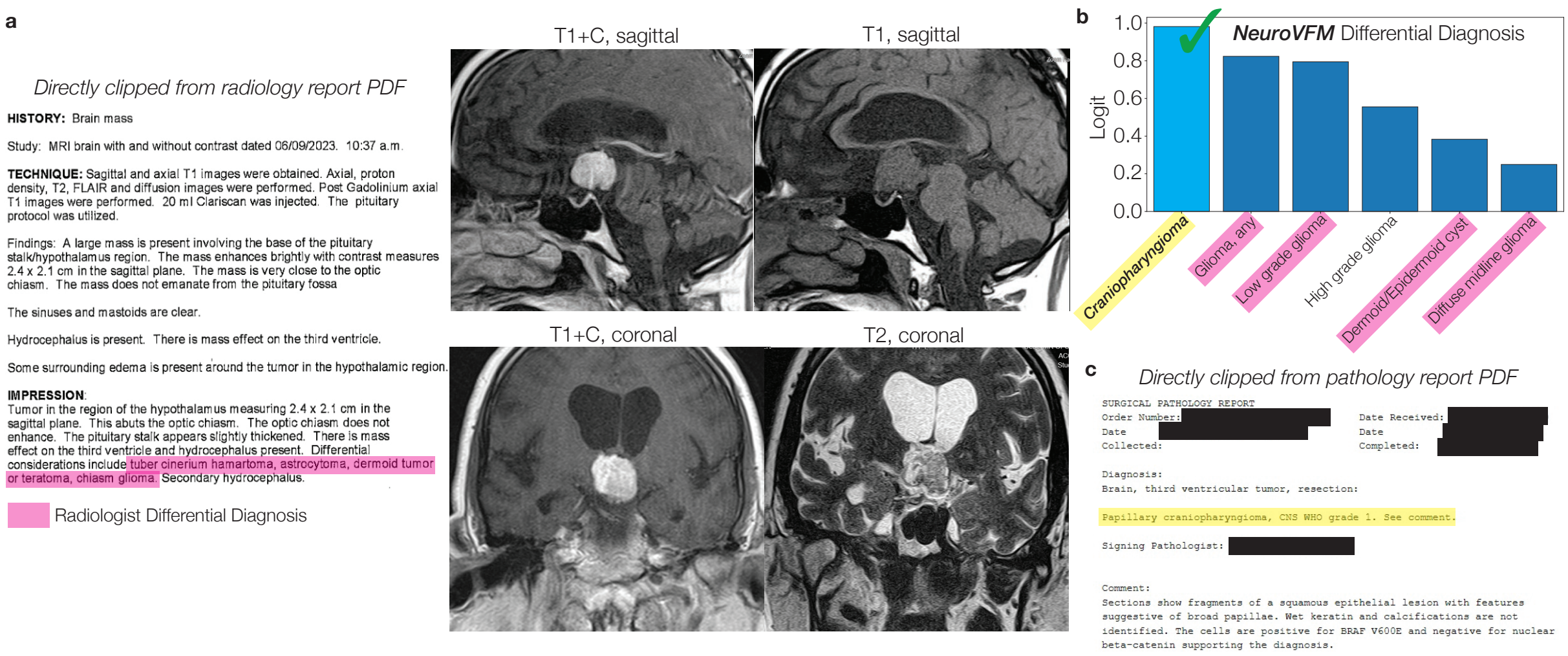

Supplementary Data Figure 3 | **Radiologic error case study.** **a**, We present an illustrative case that demonstrates a radiologist's interpretation error (Renfrew class II error: faulty reasoning), such that an abnormality was identified but attributed to the wrong cause. The correct diagnosis, craniopharyngioma, was not included in the radiology report, despite the attending radiologist providing a broad differential diagnostic list. Our neuro-radiology co-authors (V.G., A.S.) believe this represents a significant error after their review. **b**, NeuroVFM correctly assigned the highest probability to craniopharyngioma in the differential diagnosis list. **c**, Final diagnosis was confirmed as papillary craniopharyngioma.

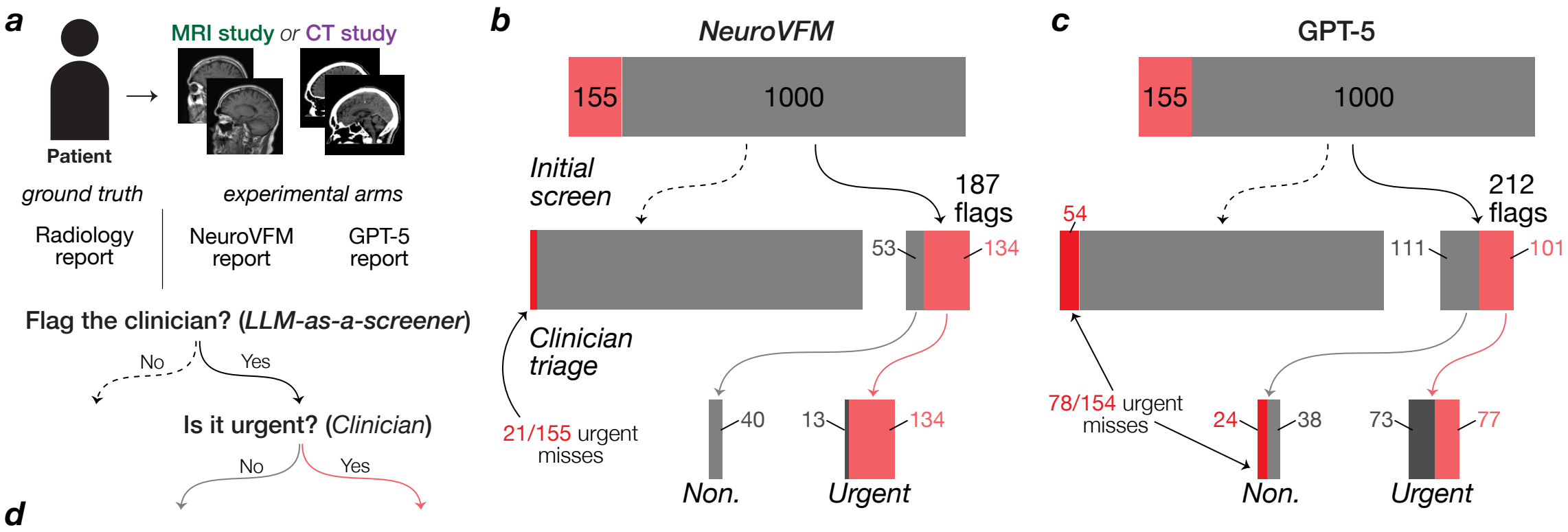

d

Prompt: Flag study given neuroimaging study, clinical indication, and list of critical findings

```
# Role:
You are an expert neuro-radiologist AI assistant.
# Objective:
Analyze the provided list of radiological findings from a neuroimaging study, using the provided clinical indication to inform your analysis, and determine if the case requires urgent clinical attention based on a strict set of criteria.
# Instructions:
1. Assess: Write a single, concise "urgency_assessment" sentence explaining whether the findings represent an urgent condition based strictly on the provided criteria below.
2. Classify: Determine if the case is "Urgent" ('true') or "Not Urgent" ('false').
# Urgency Criteria:
A study is classified as Urgent ('is_urgent: true') ONLY if it contains one or more of the following specific findings:
1. Acute/subacute Hemorrhage/Hematoma: Any acute/subacute epidural, subdural, intraparenchymal, subarachnoid, intraventricular, or intramedullary hemorrhage.
2. Acute Stroke or Ischemia: Evidence of acute/subacute ischemic infarction 3. Brain Tumor or Mass: Presence of intracranial mass or tumor. 4. Brain Herniation: Any brain herniation (e.g., subfalcine, uncal) EXCLUDING tonsillar due to Chiari malformation. 5. Hydrocephalus: Marked or acute (e.g., transependymal flow) enlargement of the ventricles. 6. CNS abscess: Clear radiographic evidence of intracranial abscess.\ 7. Catheter Malposition: A surgical catheter (e.g., EVD, shunt) that is misplaced or malpositioned.
8. Mass Effect: Substantial displacement of brain structures due to a mass, hemorrhage, or edema.
9. Severe Skull or Facial Fracture: Depressed skull fracture, open skull fracture, orbital fractures, facial fractures.
10. Metastatic Disease: Evidence of diffuse metastatic lesions.
If none of these specific criteria are met, the study should be classified as Not Urgent ('is_urgent: false').
# Urgency Exceptions
These cases should be considered NOT urgent:
- Chiari malformations alone are not urgent, but are urgent if associated with spine syrinx.
- Tonsillar herniation/ectopia due to Chiari malformation.
- Small volume hemorrhage/hematoma, minor postoperative hemorrhage.
- Small regions of diffusion restriction.
- Minor signs of elevated intracranial pressure due to idiopathic intracranial hypertension.
# Output Format
- JSON object with 2 fields:
{
  "urgency_assessment": "...",
  "is_urgent": true/false
}
Now, evaluate the given radiological findings and clinical indication against the urgency criteria.
```

Supplementary Data Figure 4 | **Expanded prospective evaluation of report sufficiency for triage. a**, Schematic of how the prospective study was set up. All neuroimaging studies satisfying our inclusion criteria with a paired radiology report were acquired during a 1-week consecutive window from January 18th-25th, 2026. For all studies, we generated two reports using NeuroVFM-LLaVA and GPT-5. First, reports (with the associated clinical indication) were flagged if suspected to have at least one critical finding by a fixed screening model. If flagged, an expert clinician then manually reviewed the report, assigned a final, gold-standard triage decision. **b-c**, Flowcharts of the outcomes of the prospective study for NeuroVFM-LLaVA and GPT-5. **d**, Prompt used to flag studies by the screening model. The list of critical findings was limited to cranial neuroradiology and informed by (18).

SQL Query: Retrieve neuroimaging studies from PACS

```
USE "UMHS SDWH Datamodel";
GO

SELECT ExaminationDate, ExaminationInstitution, ExaminationAccessionNumber, ExaminationPatientId, ExaminationBodyPart,
        ExaminationModality, ExaminationDescription, Patients.PatientPersonalId, Patients.PatientBirthdate, patients.PatientSex,
        patients.PatientDeceased,Reports.ReportText, Reports.ReportDate


FROM dbo.Examinations INNER JOIN dbo.Patients ON Patients.PatientId = Examinations.ExaminationPatientId
    inner join dbo.ExaminationReports on ExaminationReports.ExaminationReportExaminationId = Examinations.ExaminationId
    inner join dbo.Reports on Reports.ReportId = ExaminationReports.ExaminationReportReportId

WHERE (Examinations.ExaminationBodyPart like '%Brain%' or Examinations.ExaminationBodyPart like '%Head%'
or Examinations.ExaminationBodyPart like '%Orbit%' or Examinations.ExaminationBodyPart like '%Neck%'
or Examinations.ExaminationDescription like '%Brain%' or Examinations.ExaminationDescription like '%Head%'
or Examinations.ExaminationDescription like '%Orbit%' or Examinations.ExaminationDescription like '%Neck%')
AND Examinations.ExaminationDate <= '2023-06-01 00:00:00'
```

Supplementary Data Figure 5 | **SQL query to retrieve neuroimaging studies**. Using the SDW, we retrieved all CT and MRI studies whose body part or description contained "brain," "head," "orbit(s)," or "neck," returning exam metadata and report text. The SQL query filters studies acquired before June 1, 2023, for model development. The temporally held-out test set applies the same query on and after that date.

Prompt: parse MRI diagnoses from report

```
You, ChatGPT, are an expert neuroradiologist specializing in the interpretation of brain and spine MRI imaging reports. You will classify a radiology report according to the predefined list diagnoses below. This is a multilabel classification task, meaning a patient may have any number of these diagnoses (including none).

Your job is to:
1. Parse the free-text radiology report.
2. Determine the presence or absence of each diagnosis based strictly on the imaging findings and/or impression sections.
3. Output a structured JSON object where:
  - Each diagnosis is a key.
  - The value is an object with:
  - "rationale": A one-sentence explanation of the decision.
  - "label": "yes" if the diagnosis is present based on imaging findings and/or impression, "no" otherwise.

Strict Classification Guidelines:
- ONLY base the classification on the FINDINGS and IMPRESSION sections.
  - Ignore the INDICATION/HISTORY section unless it directly correlates with imaging findings.
  - A history of a condition does NOT mean it is present in the current imaging.
- A diagnosis should be labeled `"yes"` only if explicitly stated in the imaging findings or mentioned as a possible diagnosis.
  - Example: "There is evidence of..." -> `"yes"`
  - Example: "Findings are consistent with..." -> `"yes"`
  - Example: "Diagnoses on the differential are..." -> "yes"
- A diagnosis must be labeled `"no"` if the report explicitly negates it or states it is resolved.
  - Example: "No evidence of..." -> `"no"`
  - Example: "Previously resected..." -> `"no"`
  - Example: "Normal exam, no abnormal findings..." -> `"no"`
- If a condition is not mentioned at all in the findings/impression, classify it as `"no"`** by default.
  - Example: If "brain metastasis" is not discussed in the findings, set `"brain_metastasis": {"rationale": "No mention of brain metastasis in
    the findings.", "label": "no"}`
- If the report states that there is no residual or no recurrence of a prior diagnosis, classify it as `"no"`.
  - Example: "No residual tumor seen." -> `"no"`
  - Example: "Prior infarct, but no acute ischemic stroke" -> `"acute_ischemic_stroke": "no"`

Expected JSON Output Format

{
  "subdural_hematoma": {
    "rationale": "No mention of subdural hematoma is present in the report.",
    "label": "no"
  },
  "epidural_hematoma": {
    "rationale": "The report explicitly states there is no epidural hematoma.",
    "label": "no"
  },
  "encephalomalacia": {
    "rationale": "The report describes hypodense areas consistent with prior infarction, suggesting encephalomalacia.",
    "label": "yes"
  },
  "...": {
    "rationale": "...",
    "label": "..."
  }
}

Diagnoses List (Ensure a Key for Each in the JSON Output):
<diagnoses, refer to caption>

Final Notes:
- Every diagnosis must have a key in the JSON output.
- Ensure strict adherence to the format (no missing brackets, misplaced commas, colons, or inconsistent tuple usage).
- Avoid free-text responses; only structured JSON format is allowed.
```

Supplementary Data Figure 6 | **MRI diagnosis extraction prompt.** Caption on next page.

Supplementary Data Figure 6 | **MRI diagnosis extraction prompt.** We used an LLM-based annotation pipeline (4) with GPT-4.1-mini to convert free-text radiology reports into structured labels for 74 expert-defined MRI diagnoses. The prompt requires, for each diagnosis, a present/absent label and a brief rationale supporting the decision. The full list of MRI diagnoses is: subdural_hematoma; epidural_hematoma; brain_contusion; diffuse_axonal_injury; cerebral_aneurysm; arteriovenous_malformation; cavernous_malformation_cavernoma ("yes" if radiologist confident for cavernoma; "no" if possible microbleed); venous_sinus_thrombosis; acute_ischemic_stroke ("yes" for acute ischemia/infarct with diffusion restriction); chronic_ischemic_stroke ("yes" for chronic or evolving ischemia/infarct without diffusion restriction); moya_moya_disease; small_vessel_ischemic_disease (also known as microvascular ischemic disease); lacunar_stroke; intracranial_hemorrhage ("yes" for any intracranial hemorrhage, excluding microhemorrhage); intraparenchymal_hemorrhage ("yes" for hemorrhages within the brain, usually spontaneous and due to hypertension); intraventricular_hemorrhage; aneurysmal_subarachnoid_hemorrhage ("yes" if due to ruptured aneurysm or other vascular cause); traumatic_subarachnoid_hemorrhage ("yes" if due to head trauma); multiple_sclerosis; neuromyelitis_optica; neurosarcoidosis; brain_abscess ("yes" if radiologist is confident for brain abscesses or subdural empyemas); viral_encephalitis (most commonly herpes encephalitis involving the temporal and frontal lobes); arachnoid_cyst; pineal_cyst; epidermoid_cyst ("yes" for intracranial epidermoid cyst only); colloid_cyst; brain_tumor ("yes" for any intracranial mass, growth, or neoplasm); orbital_tumor ("yes" for any orbital mass, growth, or neoplasm); head_neck_tumor ("yes" for tumor/cancer in the oral cavity, throat, larynx, nasal cavity, salivary, and thyroid gland); spine_tumor ("yes" for any spine mass, growth, or neoplasm); nerve_tumor ("yes" for nerve, nerve root, or nerve sheath tumors); spinal_degenerative_changes; glioma ("yes" if mostly likely primary glial tumor); high_grade_glioma ("yes" if MRI features for high-grade glioma); low_grade_glioma ("yes" if MRI features for low-grade glioma); brain_metastasis; meningioma; schwannoma; lymphoma; pineal_tumor ("yes" for any tumor in the pineal region); intraventricular_tumor; pediatric_posterior_fossa_tumor (diagnosis includes, but not limited to, medulloblastoma, ependymoma, pilocytic astrocytoma); brainstem_glioma (diagnosis also called diffuse midline gliomas); pituitary_tumor; rathkes_cleft_cyst; craniopharyngioma; chordoma_chondrosarcoma; chiari_malformation ("yes" for chiari I/II malformations); cerebral_atrophy; brain_herniation; brain_mass_effect; brain_midline_shift; cephaloceles; encephalomalacia_gliosis; cerebral_edema; cavum_septum_pellucidum; dandy_walker_malformation; mega_cisterna_magna; heterotopia; pachygyria_lissencephaly; schizencephaly; dysgenesis_corpus_callosum; ventriculomegaly ("yes" for any abnormally enlarged ventricles); hydrocephalus_ex_vacuo ("yes" for ventriculomegaly due to brain atrophy, normal pressure hydrocephalus, or ex vacuo dilation); obstructive_hydrocephalus ("yes" for ventriculomegaly due to mass lesion or aqueductal stenosis); subdural_hygroma_effusions; intracranial_hypotension; craniotomy_craniectomy; tumor_resection_cavity; postsurgical_changes; pneumocephalus; catheter ("yes" for any intracranial catheter); spine_syrinx.

Prompt: parse CT diagnoses from report

```
You, ChatGPT, are an expert neuroradiologist specializing in the interpretation of brain and spine CT imaging reports. You will classify a
radiology report according to the predefined list diagnoses below. This is a multilabel classification task, meaning a patient may have any
number of these diagnoses (including none).

Your job is to:
1. Parse the free-text radiology report.
2. Determine the presence or absence of each diagnosis based strictly on the imaging findings and/or impression sections.
3. Output a structured JSON object where:
  - Each diagnosis is a key.
  - The value is an object with:
  - "rationale": A one-sentence explanation of the decision.
  - "label": "yes" if the diagnosis is present based on imaging findings and/or impression, "no" otherwise.

Strict Classification Guidelines:
- ONLY base the classification on the FINDINGS and IMPRESSION sections.
  - Ignore the INDICATION/HISTORY section unless it directly correlates with imaging findings.
  - A history of a condition does NOT mean it is present in the current imaging.
- A diagnosis should be labeled `"yes"` only if explicitly stated in the imaging findings or mentioned as a possible diagnosis.
  - Example: "There is evidence of..." -> `"yes"`
  - Example: "Findings are consistent with..." -> `"yes"`
  - Example: "Diagnoses on the differential are..." -> "yes"
- A diagnosis must be labeled `"no"` if the report explicitly negates it or states it is resolved.
  - Example: "No evidence of..." -> `"no"`
  - Example: "Previously resected..." -> `"no"`
  - Example: "Normal exam, no abnormal findings..." -> `"no"`
- If a condition is not mentioned at all in the findings/impression, classify it as `"no"`** by default.
  - Example: If "brain metastasis" is not discussed in the findings, set `"brain_metastasis": {"rationale": "No mention of brain metastasis in
    the findings.", "label": "no"}`
- If the report states that there is no residual or no recurrence of a prior diagnosis, classify it as `"no"`.
  - Example: "No residual tumor seen." -> `"no"`
  - Example: "Prior infarct, but no acute ischemic stroke" -> `"acute_ischemic_stroke": "no"`

Expected JSON Output Format

{
  "acute_subdural_hematoma": {
    "rationale": "No mention of acute subdural hematoma is present in the report.",
    "label": "no"
  },
  "epidural_hematoma": {
    "rationale": "The report explicitly states there is no epidural hematoma.",
    "label": "no"
  },
  "encephalomalacia": {
    "rationale": "The report describes hypodense areas consistent with prior infarction, suggesting encephalomalacia.",
    "label": "yes"
  },
  "...": {
    "rationale": "...",
    "label": "..."
  }
}

Diagnoses List (Ensure a Key for Each in the JSON Output):
<diagnoses, refer to caption>

Final Notes:
- Every diagnosis must have a key in the JSON output.
- Ensure strict adherence to the format (no missing brackets, misplaced commas, colons, or inconsistent tuple usage).
- Avoid free-text responses; only structured JSON format is allowed.
```

Supplementary Data Figure 7 | **CT diagnosis extraction prompt**. Caption on next page.

Supplementary Data Figure 7 | **CT diagnosis extraction prompt.** We used an LLM-based annotation pipeline (4) with GPT-4.1-mini to convert free-text radiology reports into structured labels for 82 expert-defined CT diagnoses. The prompt requires, for each diagnosis, a present/absent label and a brief rationale supporting the decision. The full list of CT diagnoses is here:
acute_subdural_hematoma ("yes" if acute blood in subdural space, often after head trauma and associated with midline shift); subacute_chronic_subdural_hematoma ("yes" if subacute or chronic blood in the subdural space, often non-traumatic); epidural_hematoma; brain_contusion; displaced_skull_fracture ("yes" if displaced or depressed skull fracture); nondisplaced_skull_fracture; skull_base_fracture; orbital_trauma ("yes" if orbit fracture, globe rupture, or other orbital trauma); orbital_emphysema; pneumocephalus; diffuse_axonal_injury; craniofacial_injury ("yes" if any mandibular, maxillary, zygoma, or other facial fractures or trauma); intracranial_hemorrhage ("yes" any intracranial hemorrhage); intraparenchymal_hemorrhage ("yes" for hemorrhages within the brain, usually spontaneous and due to hypertension); aneurysmal_subarachnoid_hemorrhage ("yes" if due to ruptured aneurysm or other vascular cause); traumatic_subarachnoid_hemorrhage ("yes" if due to head trauma); intraventricular_hemorrhage; scalp_hemorrhage_hematoma; acute_ischemic_stroke ("yes" for acute ischemia/infarct often with cerebral edema, mass effect, midline shift); small_vessel_ischemic_disease (also known as microvascular ischemic disease); large_vessel_occlusion ("yes" if large-vessel occlusion found on CT angiogram); intracranial_atherosclerosis; arterial_dissection ("yes" if dissection found on CT angiography); carotid_cavernous_fistula; cerebral_venous_sinus_thrombosis ("yes" if thrombosis found on CT angiogram/venogram); intracranial_aneurysm ("yes" if found on CT angiogram); cavernous_malformation_cavernoma ("yes" if radiologist confident for cavernoma; "no" if possible microbleed); cerebral_arteriovenous_malformation; intra_axial_brain_tumor ("yes" for tumors within the brain parenchyma); extra_axial_brain_tumor ("yes" for tumors outside the brain parenchyma, but inside the skull/intracranial space); orbital_tumor ("yes" if diagnosis of any orbital mass, growth, or neoplasm); pineal_tumor ("yes" for any tumor in the pineal region); pituitary_tumor; skull_bone_tumor; posterior_fossa_tumors ("yes" for any tumor in the posterior fossa, cerebellum, or brainstem); head_neck_tumor ("yes" if diagnosis of tumor/cancer in the oral cavity, throat, larynx, nasal cavity, salivary, and thyroid gland); spine_tumor ("yes" if diagnosis of any spine mass, growth, or neoplasm); spinal_degenerative_changes; arachnoid_cyst; intracranial_epidermoid_dermoid_cyst; colloid_cyst; cerebral_subdural_empyema; cerebral_abscess; craniotomy; cranioplasty_implant ("yes" if artificial implant to repair skull defect); burr_hole; resection_cavity; postsurgical_changes; catheter ("yes" for any intracranial catheter); intracranial_pressure_monitor; brain_lead ("yes" for brain electrode leads used in functional neurosurgery); aneurysm_coil; aneurysm_clip; embolization_material; cerebral_foreign_body (examples include glass, metal, plastic, wood); ventriculomegaly; obstructive_hydrocephalus; transependymal_flow; subdural_hygroma_effusions; slit_ventricle; intracranial_hypotension; cerebral_edema; cerebral_atrophy; brain_herniation; brain_mass_effect; midline_shift; encephalomalacia_gliosis; basal_ganglia_calcification; chiari_malformation ("yes" for Chiari I/II malformations); dysgenesis_corpus_callosum; dandy_walker_malformation; mega_cisterna_magna; sinusitis; mastoid_effusion (includes mastoid fluid and mastoiditis); otitis_media; peritonsillar_abscess; pharyngeal_abscess (includes retropharyngeal and parapharyngeal abscesses); cholesteatoma; head_neck_enlarged_lymph_node; airway_obstruction (examples include septal deviation, choanal atresia, obstruction, airway edema, laryngeal edema, and pharyngeal edema); thyroid_nodule; parathyroid_nodule.

Step 1: Summarize findings from report

Your task is to shorten MRI reports by summarizing abnormalities and removing certain information. Please summarize the abnormalities in the report above in an one-level enumerated list without indents. Only include abnormalities. Your summarization must not include items that are normal or not evident (like "No ...", or "No evidence of ...", or "Normal ..." or "Unremarkable ..."). If there is no abnormalities in this report, write "1. Study is unremarkable."

Step 2: Remove unremarkable statements

Your goal is to modify a list of radiology findings with the following rules:
1. Remove all the items in the list that are fully normal (i.e. items that indicates something is normal or unremarkable, or nothing special is found, or no evidence or nothing significant). Write "<Removed>" for the removed items.
2. If an item in the list contains both normal and abnormal information, only remove the normal parts. Keep the abnormal parts in the item, and do not delete the item. Make sure you remove sentences staring with "No ..." or "Unremarkable ...".
3. Please also remove all phrases that contain "clinical correlation" or suggestions for further evaluation with additional studies (such as recommending future MRIs).
4. Do not remove other abnormal information unless specified above. Do not remove benign abnormalities.

Step 3: Remove comparisons to prior studies and measurements

Your goal is to modify a list of radiology findings with the following rules:
1. Remove all phrases that indicates a comparison to a previous report, such as redemonstration, stable, stable in comparison, unchanged, compared to, prior exam, interval improvement, interval worsening, interval increase, interval reduction, old, increase in size, reduction in size, etc.
2. Please also remove all numerical size measurements, such as "8 mm" or "3 cm". Do not remove non-numerical description of size, such as "small", "minimal" or "large".
3. Please remove all date phrases such as "06/20/2012" from all items.\n\n4. Please remove all references to patient history, such as "compatible with history of ..."

Your output should have the same number of rows as the input.

Supplementary Data Figure 8 | **Prompts for structured findings extraction.** Three successive prompts were applied to GPT-4.1-mini to turn free-text radiology reports into an itemized list of structured findings for report generation. The pipeline extracts key positive findings and removes unremarkable statements, comparisons to prior studies, and raw measurements. The resulting findings supervised training of NeuroVFM-LLaVA for preliminary findings generation.

Prompt: Generate findings given neuroimaging study and clinical indication

```
# Objective:
Analyze the provided neuroimaging study, using the provided clinical indication to inform your analysis, and generate a structured findings report as a single JSON object.

# Instructions:
Assess the images and list all relevant radiological findings, using the Findings List Guidelines below. NOTE: The provided images are cross-sectional slices from one or more 3D scans from the study. **Images are presented in radiological orientation, where the LEFT side of the image corresponds to the RIGHT side of the patient.**

# Findings List Guidelines:
- **Analyze the Provided Images Only:** All findings must be based on the provided image only. Do NOT make statements that imply comparison to a prior study (e.g., 'stable/progressing', 'growing/shrinking').
- **Include Significant Pertinent Positives:** The list must only contain statements about the existence of abnormal, clinically significant findings. If there are no abnormal findings, you must include a single item in the list "Study is unremarkable."
- **Exclude Negative/Normal Findings:** Do **not** include statements about the absence of a pathology (e.g., 'no hemorrhage') or statements about normalcy of structures (e.g., 'ventricles are normal').
- **Direct Language:** Rewrite findings as direct anatomical descriptions. Remove introductory phrases.

# Output Format
- Your response must consist of ONLY a single JSON object with a single field "findings", which should be a list of strings
{
  "findings": [
    "...",
    "...",
    "...",
    ...
  ]
}

Now, analyze the following neuroimaging study in the context of the given indication.
```

Supplementary Data Figure 9 | **Frontier model prompt for study findings.** To assess reasoning models (e.g., GPT-5-thinking) on preliminary neuroimaging findings generation, we passed the clinical indication and the study slice-by-slice. The prompt asks for an itemized list of key findings at the study level.

Supplementary Data Table 1 | **Expert validation results of LLM annotation pipeline.** The table lists the full results of the expert analysis of a stratified subset of LLM-derived diagnostic annotations in UM-NeuroImages, grouped by modality and diagnostic category. *This large table is available in the accompanying spreadsheet.*

Supplementary Data Table 2 | **Descriptive characteristics of the UM-NeuroImages test set.** The temporally held-out UM-NeuroImages test set comprises 50,293 CT and MRI studies. For each study, the table reports modality, scanner manufacturer, acquisition site, patient age at scan (with ages ⩾90 years truncated to 90 years due to PHI constraints), sex, ethnicity, and MRI field strength (when applicable). *This large table is available in the accompanying spreadsheet.*

Supplementary Data Table 3 | **Expanded results for UM-NeuroImages 156 diagnostic tasks.** The table lists an expanded suite of results for the 156 diagnostic results for NeuroVFM and four baselines (HLIP, NeuroMAE, DINOv3, BiomedCLIP), including precision, recall, F1, and sensitivity at 90% specificity. *This large table is available in the accompanying spreadsheet.*

Supplementary Data Table 4 | **Expanded results of category-level comparisons for UM-NeuroImages corrected for multiple hypotheses.** The table lists paired bootstrap results between NeuroVFM and all comparators for both CT and MRI, corrected for multiple hypotheses using Benjamini-Hochberg correction with a false discovery rate of 0.05. *This large table is available in the accompanying spreadsheet.*

Supplementary Data Table 5 | **Performance of NeuroVFM on external neuroimaging benchmarks.** The table summarizes the performance of NeuroVFM and four baseline models (HLIP, NeuroMAE, DINOv3, and BiomedCLIP) on eight external neuroimaging benchmarks (six MRI and two CT datasets). All models were evaluated using 8-fold stratified cross-validation with a 20% held-out test split, except for AIBL and OASIS-1, which were evaluated on the entire dataset using the ADNI-trained classifier. *This large table is available in the accompanying spreadsheet.*

Supplementary Data Table 6 | **Generated reports and evaluations for the UM-NeuroImages-Acuity test set.** For the balanced UM-NeuroImages-Acuity test set of 300 studies, the table includes generated reports from all models, the corresponding ground-truth radiology reports, clinical indication, acuity labels, and all associated evaluation scores from expert raters. *This large table is available in the accompanying spreadsheet.*

Supplementary Data Table 7 | **Generated reports and evaluations for prospective evaluation of report sufficiency.** For the 1-week prospective cohort, the table lists generated reports from all models, the corresponding ground-truth radiology reports, clinical indication, triage labels, and flagging status with the screening model. *This large table is available in the accompanying spreadsheet.*

Supplementary Data Table 8 | **Expanded results of the prospective triage feasibility study.** The table lists the balanced accuracy, sensitivity, specificity, positive predictive value (PPV), and negative predictive value (NPV) for clinical triage performance using either NeuroVFM-generated or GPT-5-generated reports. Metrics are reported with a 95% bootstrapped CI (10,000 replicates). *This large table is available in the accompanying spreadsheet.*

Supplementary Data Table 9 | **Hyperparameters for NeuroVFM pretraining and UM-NeuroImages study-level attentive probing.** The table lists all hyperparameters used to (1) pretrain the 3D ViT backbone and (2) train the study-level attentive probe used for UM-NeuroImages. For each configuration, we report optimization settings, data sampling strategies, architectural choices, and regularization parameters. A similar probing strategy was used for all external benchmarks, with hyperparameters adjusted for dataset size and complexity. *This large table is available in the accompanying spreadsheet.*

Supplementary Data Table 10 | **Full task-level performance on the UM-NeuroImages CT test set.** The temporally held-out UM-NeuroImages CT test set comprises 21,054 CT studies with 82 study-level CT diagnoses. For each diagnosis and diagnostic category, the table reports the classification performance of NeuroVFM and three baseline models (HLIP, NeuroMAE, DINOv3, and BiomedCLIP). *This large table is available in the accompanying spreadsheet.*

Supplementary Data Table 11 | **Full task-level performance on the UM-NeuroImages MRI test set.** The temporally held-out UM-NeuroImages MRI test set comprises 29,239 MRI studies with 74 study-level MRI diagnoses. For each diagnosis and diagnostic category, the table reports the classification performance of NeuroVFM and five baseline models (HLIP, NeuroMAE, DINOv3, BiomedCLIP, and PRIMA). *This large table is available in the accompanying spreadsheet.*

Supplementary Data Table 12 | **New-patient test set results.** The table lists the AUROCs of NeuroVFM and four baselines (HLIP, NeuroMAE, DINOv3, BiomedCLIP) on the test set with overlapping patients removed. Aggregate performance on MRI drops by 0.7-1.1 AUROC points, with model rankings unchanged. *This large table is available in the accompanying spreadsheet.*

Supplementary Data Table 13 | **Hyperparameters for NeuroVFM-LLaVA training and inference.** The table lists all hyperparameters used to perform LLaVA-1.5-style vision-instruction tuning on top of the frozen NeuroVFM backbone to finetune a language model for key findings generation. Reported settings include model initialization, optimizer configuration, learning rate schedule, batch size, context length, training duration, and inference parameters. The deployed inference pipeline is implemented with HuggingFace to enable standardized report generation. *This large table is available in the accompanying spreadsheet.*
