# Supplementary material for: Health system learning enables generalist neuroimaging models
Source: Nat Med. 2026 Jul 10;32(8):2831–7. doi: 10.1038/s41591-026-04497-1 (PMC13472962; doi:10.1038/s41591-026-04497-1)
Supplement: Supplementary file 2 [file 41591_2026_4497_MOESM2_ESM.pdf]

nature portfolio

Corresponding author(s): Todd Hollon, MD

Last updated by author(s): 11-16-2025

Reporting Summary

Nature Portfolio wishes to improve the reproducibility of the work that we publish. This form provides structure for consistency and transparency in reporting. For further information on Nature Portfolio policies, see our Editorial Policies and the Editorial Policy Checklist.

Statistics

For all statistical analyses, confirm that the following items are present in the figure legend, table legend, main text, or Methods section.

n/a	Confirmed	
☐	☒	The exact sample size (*n*) for each experimental group/condition, given as a discrete number and unit of measurement
☒	☐	A statement on whether measurements were taken from distinct samples or whether the same sample was measured repeatedly
☐	☒	The statistical test(s) used AND whether they are one- or two-sided<br>*Only common tests should be described solely by name; describe more complex techniques in the Methods section.*
☒	☐	A description of all covariates tested
☐	☒	A description of any assumptions or corrections, such as tests of normality and adjustment for multiple comparisons
☐	☒	A full description of the statistical parameters including central tendency (e.g. means) or other basic estimates (e.g. regression coefficient) AND variation (e.g. standard deviation) or associated estimates of uncertainty (e.g. confidence intervals)
☐	☒	For null hypothesis testing, the test statistic (e.g. *F*, *t*, *r*) with confidence intervals, effect sizes, degrees of freedom and *P* value noted<br>*Give P values as exact values whenever suitable.*
☒	☐	For Bayesian analysis, information on the choice of priors and Markov chain Monte Carlo settings
☒	☐	For hierarchical and complex designs, identification of the appropriate level for tests and full reporting of outcomes
☐	☒	Estimates of effect sizes (e.g. Cohen's *d*, Pearson's *r*), indicating how they were calculated

Our web collection on statistics for biologists contains articles on many of the points above.

Software and code

Policy information about availability of computer code

Data collection	Data was collected as part of routine clinical care at the University of Michigan health system. Data was queried from the picture archive and communications system (PACS) via Sectra Data Warehouse for neuroimaging studies within the premises of our IRB.
Data analysis	All code was implemented in Python (version 3.10.14) using PyTorch Lightning (2.5.0.post0) compiled with CUDA 12.4 as the primary machine learning framework. The following packages were used for data preprocessing, model training and evaluation: pydicom (2.4.4), nibabel (5.3.2), SimpleITK (2.4.0), torchvision (0.20.0), pandas (2.2.3), NumPy (2.1.2), flash_attn (2.6.3), matplotlib (3.10.7), scipy (1.15.2), and scikit-learn (1.6.1). The following packages were used to load baselines: open_clip (2.23.0) and transformers (4.56.0). All code and scripts to reproduce the experiments of this paper are available on GitHub at https://github.com/MLNeurosurg/neurovfm under an MIT license.

For manuscripts utilizing custom algorithms or software that are central to the research but not yet described in published literature, software must be made available to editors and reviewers. We strongly encourage code deposition in a community repository (e.g. GitHub). See the Nature Portfolio guidelines for submitting code & software for further information.

Data

Policy information about availability of data

All manuscripts must include a data availability statement. This statement should provide the following information, where applicable:

- Accession codes, unique identifiers, or web links for publicly available datasets
- A description of any restrictions on data availability
- For clinical datasets or third party data, please ensure that the statement adheres to our policy

Institutional Review Board approval was obtained from University of Michigan for MRI data collection. Restrictions apply to the availability of raw patient MRI imaging data, which were used with institutional permission through IRB approval for the current study, and are thus not publicly available. All data sharing between medical centers is regulated through data use agreements with the study authors. A similar data-sharing protocol may be established for interested investigators. Please contact the corresponding author (T.H.) for any requests for data sharing. All requests will be evaluated based on institutional and departmental policies to determine whether the data requested is subject to intellectual property or patient privacy obligations. Data can only be shared for non-commercial academic and investigational purposes.

Research involving human participants, their data, or biological material

Policy information about studies with human participants or human data. See also policy information about sex, gender (identity/presentation), and sexual orientation and race, ethnicity and racism.

Reporting on sex and gender	We report per-modality subgroup performance by sex (attributed at birth) in our prospective set in Extended Data Fig. 6.
Reporting on race, ethnicity, or other socially relevant groupings	We report per-modality subgroup performance by ethnicity in our prospective set in Extended Data Fig. 6.
Population characteristics	The retrospective set used to train and validate NeuroVFM spans more than 20 years of routine clinical care at the University of Michigan health system, representative of the state-wide population at large. We include a descriptive breakdown of this training set by volume, study, and patient in Extended Data Fig. 2. We also include the full information per-study of our prospective test set (21,054 CT and 29,239 MRI studies) in Supplementary Data Table 1.
Recruitment	Neuroimaging studies that satisified our criteria were retrospectively queried from the PACS. A temporally held-out prospective set was acquired in a similar fashion between June 1, 2023 and May 30, 2024.
Ethics oversight	Our research was approved by the University of Michigan Institutional Review Board (HUM00229133). All MRI data was acquired under secondary data usage. The methods were carried out in accordance with the IRB's guidelines, regulations, and policies. All human subjects that met inclusion criteria as stated above were included in the study.

Note that full information on the approval of the study protocol must also be provided in the manuscript.

Field-specific reporting

Please select the one below that is the best fit for your research. If you are not sure, read the appropriate sections before making your selection.

☒ Life sciences ☐ Behavioural & social sciences ☐ Ecological, evolutionary & environmental sciences

For a reference copy of the document with all sections, see nature.com/documents/nr-reporting-summary-flat.pdf

Life sciences study design

All studies must disclose on these points even when the disclosure is negative.

Sample size	The prospective set consists of 21,054 CT and 29,293 MRI neuroimaging studies.
Data exclusions	We excluded data that were (1) non-image, (2) corrupted, or (3) embedded non-DICOM files.
Replication	Model training is purely deterministic. We noted initial seeds across all experiments and made our training scripts available in our public GitHub repository for replication.
Randomization	When conducting the expert preferences study to evaluate the quality of generated key findings, we provided a CSV file in which the model identifiers were anonymized (e.g., A and B) and the column order was randomized per-row.
Blinding	Raters were blinded to the model when providing preferences.

Reporting for specific materials, systems and methods

We require information from authors about some types of materials, experimental systems and methods used in many studies. Here, indicate whether each material, system or method listed is relevant to your study. If you are not sure if a list item applies to your research, read the appropriate section before selecting a response.

Materials & experimental systems

n/a	Involved in the study
☒	☐ Antibodies
☒	☐ Eukaryotic cell lines
☒	☐ Palaeontology and archaeology
☒	☐ Animals and other organisms
☒	☐ Clinical data
☒	☐ Dual use research of concern
☒	☐ Plants

Methods

n/a	Involved in the study
☒	☐ ChIP-seq
☒	☐ Flow cytometry
☐	☒ MRI-based neuroimaging

Plants

Seed stocks: N/A

Novel plant genotypes: N/A

Authentication: N/A

Magnetic resonance imaging

Experimental design

Design type: Data was collected during routine clinical care at the University of Michigan health system.

Design specifications: The protocol used to acquire the study was determined by the clinical team at time of acquisition.

Behavioral performance measures: N/A

Acquisition

Imaging type(s): Structural and diffusion

Field strength: Range of clinical field strengths (mostly 1.5 and 3T)

Sequence & imaging parameters: Range of sequence & imaging parameters (e.g., T1w, T2w, T2-FLAIR, T1c, DWI, etc.). Since we retrospectively collect data, all neuroimaging data is collected at the discretion of the clinical team.

Area of acquisition: All studies included contain one or more of the following areas: head, brain, orbits, face and/or neck.

Diffusion MRI: ☒ Used ☐ Not used

Parameters: Range of parameters, all at the discretion of the clinical team who requested the initial study.

Preprocessing

Preprocessing software: Minimal preprocessing was done on the raw NiFTI files acquired. We only reoriented all volumes to RPI and resampled the axis of acquisition to 4mm. Background air was masked using a Otsu-based and HU-based approach for MRI and CT, respectively, during training and inference (although not required for inference).

Normalization: We aimed to train on as much of the raw data as possible and thus decided to not normalize the data.

Normalization template: Data was not normalized.

Noise and artifact removal: No noise or artifact removal procedure was employed.

Volume censoring | N/A

Statistical modeling & inference

Model type and settings | N/A

Effect(s) tested | N/A

Specify type of analysis: ☒ Whole brain ☐ ROI-based ☐ Both

Statistic type for inference (See Eklund et al. 2016) | N/A

Correction | N/A

Models & analysis

n/a	Involved in the study
☒	☐ Functional and/or effective connectivity
☒	☐ Graph analysis
☐	☒ Multivariate modeling or predictive analysis

Multivariate modeling and predictive analysis | NeuroVFM is a 3D vision transformer trained in a self-supervised manner to generate dense representations for any clinical neuroimage. Downstream models (e.g., study-level diagnostic classifier and findings LLM) use these representations, frozen, for their respective predictive tasks. NeuroVFM's representations can be used for any task: classification, regression, segmentation, etc. We also interrogated the representations themselves by applying dimension reduction via tSNE and calculating pairwise cosine similarity after L2 normalization.
