# Supplementary material for: Health system learning enables generalist neuroimaging models
Source: Nat Med. 2026 Jul 10;32(8):2831–7. doi: 10.1038/s41591-026-04497-1 (PMC13472962; doi:10.1038/s41591-026-04497-1)
Supplement: Supplementary file 3 [file 41591_2026_4497_MOESM3_ESM.pdf]

Peer Review File

Health system learning achieves generalist neuroimaging models

Corresponding Author: Dr Todd Hollon

This file contains all reviewer reports in order by version, followed by all author rebuttals in order by version.

Version 0:

Reviewer comments:

Reviewer #1

(Remarks to the Author)
The revision is substantially improved and is highly responsive. The prospective deployment is the most important addition despite its small size. However, the prospective evaluation has gaps in metric reporting, study design (single-rater, 7-day window), and framing that should be addressed since I think it is being undersold/emphasized and is even more compelling than the authors let on (see detailed comments below).

KEY RESULTS
Same as prior revision with addition of a 1-week prospective silent deployment (N=1,155) demonstrating superior performance to GPT-5.

Validity
The prospective silent deployment (Figure 5) is a major addition that substantially strengthens the manuscript and is methodologically sound. However, there are important reporting gaps and design limitations:
(a) Missing clinical metrics. The prospective evaluation reports only balanced accuracy and critical finding miss rate. For a clinical triage evaluation, sensitivity, specificity, PPV, and NPV with 95% CIs are standard and should be reported. Based on the data in Supplementary Data Figure 7, it looks like NeuroVFM beats GPT-5 on every axis—higher sensitivity, higher specificity, higher PPV, higher NPV. This is actually a stronger result than balanced accuracy alone conveys, because it shows NeuroVFM is not simply trading sensitivity for specificity or otherwise hacking its results.
(b) Duration and generalizability. The evaluation spans 7 consecutive days at a single institution. This should be explicitly framed as a preliminary feasibility evaluation, not a validation study.
(c) Single-rater gold standard. The prospective triage gold standard was evaluated by a single clinician (T.H.) who is not a neuroradiologist and is also the senior author. While clinician blinding to report source is stated, the senior author likely can tell the difference between NeuroVFM's output patterns vs GPT-5. An external blinded neuroradiologist (ideally 3 with some form of adjudication) would have been a stronger design choice, otherwise this should be acknowledged as a major limitation.
(d) Audit of unflagged studies. The authors audited 200/969 unflagged NeuroVFM studies and found all nonurgent. However, with a 2.1% miss rate (20/969) among unflagged studies, a sample of 200 has only ~34% power to detect even a single additional miss. A larger audit or a full review of unflagged studies would provide greater confidence and seems eminently feasible. Again, this should be done or flagged as a limitation.
(e) Binary vs. 3-class triage. The prospective evaluation uses a binary triage schema (urgent vs. nonurgent, based on ASNR critical findings), whereas the balanced evaluation in Figure 4 uses a 3-class schema (unremarkable/routine/urgent). These are different tasks with different clinical semantics. The manuscript should explicitly note this difference and refrain from directly comparing the two sets of results.

The prospective GPT-5 miss rate of 53.3% (82/154 urgent cases missed) is strikingly poor. How sensitive is this to the prompting of GPT-5? How was the current prompt chosen or optimized?

Originality and Significance
All addressed

Conclusions

The discussion references “subspecialist depth, expert reliability, and safe deployment” (Lines 184–185). An 87% sensitivity for critical findings is promising but probably does not match “subspecialist depth” at this task.

The title implies generality beyond what was assessed (“health system learning achieves generalist neuroimaging models”). The model is trained on head/neck imaging from one institution, and is generally useful on this task in this institution. I feel like “generalist neuroimaging” sort of misses the point? Isn’t this more like, “best neuroimaging in-domain”?

References
Appropriately cited. No major omissions.

Clarity and Context
The manuscript is well-written and has improved since the first submission.

Assessment of My Expertise and Areas That Might Be Out of Scope
I am an expert on this topic and confident in my assessment.

(Remarks on code availability)
The code is well structured and well documented.

Reviewer #2

(Remarks to the Author)
The authors have addressed all of my comments as well as a host of other comments brought up by the other reviewers. I am overall satisfied with the current revised manuscript. One minor comment is that I do not see where Suppl Data Fig. 9 is mentioned in the manuscript. This was in response to whether NeuroVFM identified pathology potentially missed by human radiologists.

(Remarks on code availability)

Open Access This Peer Review File is licensed under a Creative Commons Attribution 4.0 International License, which permits use, sharing, adaptation, distribution and reproduction in any medium or format, as long as you give appropriate credit to the original author(s) and the source, provide a link to the Creative Commons license, and indicate if changes were made.
In cases where reviewers are anonymous, credit should be given to 'Anonymous Referee' and the source.
The images or other third party material in this Peer Review File are included in the article’s Creative Commons license, unless indicated otherwise in a credit line to the material. If material is not included in the article’s Creative Commons license and your intended use is not permitted by statutory regulation or exceeds the permitted use, you will need to obtain permission directly from the copyright holder.
To view a copy of this license, visit https://creativecommons.org/licenses/by/4.0/

Reviewer #1 (Remarks to the Author):
The revision is substantially improved and is highly responsive. The prospective deployment is the most important addition despite its small size. However, the prospective evaluation has gaps in metric reporting, study design (single-rater, 7-day window), and framing that should be addressed since I think it is being undersold/emphasized and is even more compelling than the authors let on (see detailed comments below).

KEY RESULTS
Same as prior revision with addition of a 1-week prospective silent deployment (N=1,155) demonstrating superior performance to GPT-5.

Validity
The prospective silent deployment (Figure 5) is a major addition that substantially strengthens the manuscript and is methodologically sound. However, there are important reporting gaps and design limitations:

(a) Missing clinical metrics. The prospective evaluation reports only balanced accuracy and critical finding miss rate. For a clinical triage evaluation, sensitivity, specificity, PPV, and NPV with 95% CIs are standard and should be reported. Based on the data in Supplementary Data Figure 7, it looks like NeuroVFM beats GPT-5 on every axis—higher sensitivity, higher specificity, higher PPV, higher NPV. This is actually a stronger result than balanced accuracy alone conveys, because it shows NeuroVFM is not simply trading sensitivity for specificity or otherwise hacking its results.
We agree with the reviewer and have added all relevant metrics with 95% bootstrapped CIs (10,000 replicates) for both NeuroVFM and GPT-5 in a new Supplementary Data Table 13.

**Metric (Mean, 95% CI)**	**NeuroVFM**	**GPT-5**
Balanced Accuracy	92.6% [89.8%, 95.2%]	71.2% [67.2%, 75.2%]
Sensitivity	86.5% [81.0%, 91.6%]	49.7% [41.8%, 57.6%]
Specificity	98.7% [98.0%, 99.4%]	92.7% [91.1%, 94.3%]
PPV	91.2% [86.3%, 95.5%]	51.3% [43.4%, 59.5%]
NPV	97.9% [97.0%, 98.7%]	92.2% [90.6%, 93.9%]

(b) Duration and generalizability. The evaluation spans 7 consecutive days at a single institution. This should be explicitly framed as a preliminary feasibility evaluation, not a validation study.
We agree with the reviewer. We have edited the manuscript to describe the study as a ‘preliminary feasibility evaluation’ (Line 155, 523).

(c) Single-rater gold standard. The prospective triage gold standard was evaluated by a single clinician (T.H.) who is not a neuroradiologist and is also the senior author. While clinician blinding to report source is stated, the senior author likely can tell the difference between NeuroVFM’s output patterns vs GPT-5. An external blinded neuroradiologist (ideally 3 with some form of adjudication) would have been a stronger design choice, otherwise this should be acknowledged as a major limitation.
We thank the reviewer for this comment and agree. Our rationale for having imaging-centric clinicians (e.g., neurosurgeons) assign triage labels to the reports is that they are often better able to place these radiologic findings in clinical context. We have added a blinded reviewer who is both a neurosurgeon and radiologist (A.P.). We then had a third reviewer (R.J.) adjudicate any discordant triage ground truth labels. All results have

been updated (Lines 146, 532-536). We have updated all ground-truth, GPT-5, and NeuroVFM-generated findings used in Figure 5, along with the final gold-standard triage decision, in Supplementary Data Table 9.

(d) Audit of unflagged studies. The authors audited 200/969 unflagged NeuroVFM studies and found all nonurgent. However, with a 2.1% miss rate (20/969) among unflagged studies, a sample of 200 has only ~34% power to detect even a single additional miss. A larger audit or a full review of unflagged studies would provide greater confidence and seems eminently feasible. Again, this should be done or flagged as a limitation.
We thank the reviewer for their recommendation and performed an exhaustive review of all unflagged NeuroVFM studies (N=969). We identified 1 potential study (CT_BRAIN_UM_85679BF8; Line 716 in Supplementary Data Table 9) that our initial LLM screen should have flagged. This amounts to a 99.9% sensitivity. For the final triage evaluation, we moved this study into the flagged subset to report metrics under an ideal flagging process.

(e) Binary vs. 3-class triage. The prospective evaluation uses a binary triage schema (urgent vs. nonurgent, based on ASNR critical findings), whereas the balanced evaluation in Figure 4 uses a 3-class schema (unremarkable/routine/urgent). These are different tasks with different clinical semantics. The manuscript should explicitly note this difference and refrain from directly comparing the two sets of results.
This is an astute point by the reviewer, and we have revised the manuscript to make the distinction clear. Specifically, we now refer to the clinical endpoint in Fig. 4 as ‘acuity assessment’ with the intent of evaluating report generation in a quantitative manner, and in Fig. 5 as ‘clinical triage’ with the intent of evaluating preliminary feasibility of clinical triage with NeuroVFM-generated reports.

The prospective GPT-5 miss rate of 53.3% (82/154 urgent cases missed) is strikingly poor. How sensitive is this to the prompting of GPT-5? How was the current prompt chosen or optimized?
We used the same GPT-5 prompting strategy for findings generation across Figs. 4 and 5. The prompt was chosen to optimize findings accuracy, not the detection of critical findings. Modifying the prompt to increase sensitivity would likely also increase false-positive findings, reducing specificity. In this setting, GPT-5 often failed to describe remarkable findings in urgent studies, resulting in a poor triage sensitivity.

Originality and Significance
All addressed

Conclusions
The discussion references “subspecialist depth, expert reliability, and safe deployment” (Lines 184–185). An 87% sensitivity for critical findings is promising but probably does not match “subspecialist depth” at this task.
We have removed this phrase from the sentence.

The title implies generality beyond what was assessed (“health system learning achieves generalist neuroimaging models”). The model is trained on head/neck imaging from one institution, and is generally useful on this task in this institution. I feel like “generalist neuroimaging” sort of misses the point? Isn’t this more like, “best neuroimaging in-domain”?
This is a good point by the reviewer, and we concur that ‘generalist’ in the ML literature is loosely defined. We use the ‘generalist’ term in line with the recent *Nature* review from Moor et. al., “Foundation models for generalist medical artificial intelligence”. Generalist models are ‘built through self-supervision on large, diverse datasets’, ‘used across medical applications, largely replacing task-specific models’, and ‘support flexible combinations of data modalities’. NeuroVFM was trained with self-supervision, used across several applications, and was integrated into the LLaVA architecture for multimodal input (text and images). We favor the ‘generalist’ label as NeuroVFM was not trained for any specific task, but as a general encoder of neuroradiologic data.

References
Appropriately cited. No major omissions.

Clarity and Context
The manuscript is well-written and has improved since the first submission.

Assessment of My Expertise and Areas That Might Be Out of Scope
I am an expert on this topic and confident in my assessment.

Reviewer #1 (Remarks on code availability):
The code is well structured and well documented.

Reviewer #2 (Remarks to the Author):
The authors have addressed all of my comments as well as a host of other comments brought up by the other reviewers. I am overall satisfied with the current revised manuscript. One minor comment is that I do not see where Suppl Data Fig. 9 is mentioned in the manuscript. This was in response to whether NeuroVFM identified pathology potentially missed by human radiologists.
We are delighted to see that we have addressed all comments and that the manuscript is satisfactory. We have added a reference to this figure (now Supplementary Data Fig. 7) to the manuscript (Lines 153-4).
